# Supplementary material for: Glycine, serine and threonine metabolism confounds efficacy of complement-mediated killing
Source: Nat Commun. 2019 Jul 25;10:3325. doi: 10.1038/s41467-019-11129-5 (PMC6658569; doi:10.1038/s41467-019-11129-5)
Supplement: Supplementary file 1 — Supplementary Information [file 41467_2019_11129_MOESM1_ESM.pdf]

Glycine, serine and threonine metabolism confounds efficacy of complement-mediated killing

Cheng, et al.,

Supplementary Figures

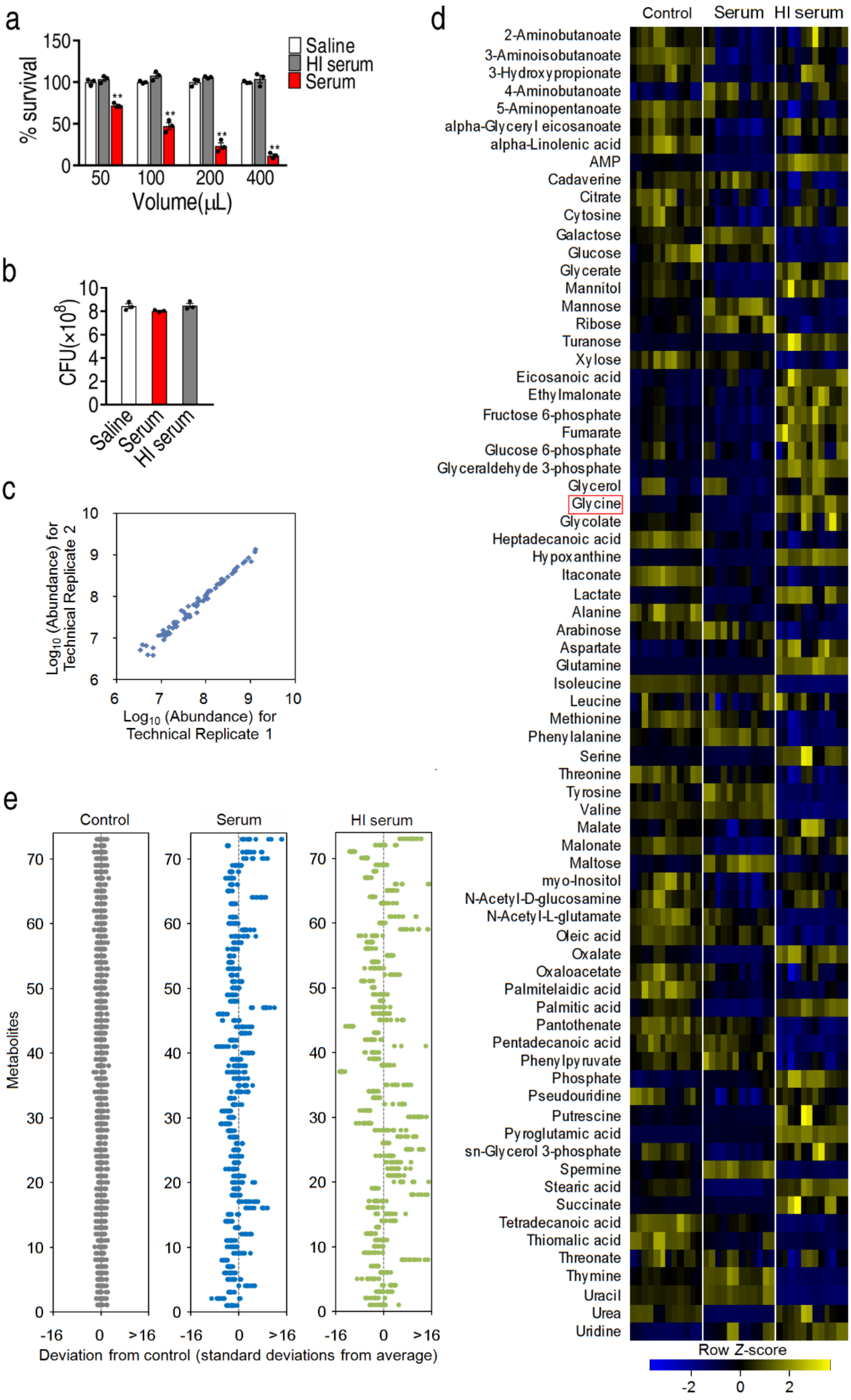

Supplementary Fig. 1 Metabolomic profile for serum-survival bacteria. a, Percent survival of *E. coli* K12 cells exposed to the indicated serum or HI serum at 37 ^{0}C for

2-h (n = 3). **b**, Bacterial number in 1 mL with 1.0 of OD600 by plate counting. K12 cells were exposed to serum, HI serum or saline. The cells were collected and washed twice using a saline buffer by centrifugation at 8,000 *g*, 5 min at 4 ^{0}C. The resulting cells were adjusted to 1.0 of OD600 and washed four times as above. Pellets were diluted for plate counting (n = 3). **c**, Reproducibility of metabolomic profiling platform used in the discovery phase. Metabolite abundances quantified in six independent biological repeats with two technical replicates were shown. Spearman correlation coefficient between technical replicates varied between 0.991 and 0.996. **d,** Heat map showing the metabolites. Yellow and blue indicated an increase and a decrease of metabolites relative to the median metabolite level, respectively (see color scale). Glycine was highlighted in the red box (n = 6). **e,** Z-score plot for data from serum-treated samples compared to the mean and standard deviation of the control samples with a Z-score range of -9.1 to 35.2. 59 out of the 73 metabolites (80.8%) were significant differential (Wilcoxon $p < 0.05$), corresponding to false discovery rate (FDR) of 9.1%. Of the 59 differential metabolites, 44 decreased and 15 increased. The Z-score plot for data from HI serum-treated cells compared to the mean and standard deviation of the control samples with a Z-score range of -14.7 to 168.9. Out of the 73 metabolites (75.3%), 55 were significantly different in abundance (Wilcoxon $P < 0.05$), corresponding to a false discovery rate (FDR) of 4.47%. Of the 55 differential metabolites, 32 decreased and 23 increased. Results (**a** and **b**) are displayed as mean ± SEM, and significant differences are identified (**$p < 0.01$) as determined by two-tailed Student's *t* test.

To identify the metabolic signature in response to serum, non-pathogenic *E. coli* K12 was treated with serum, whose percent survival was decreased with the increased serum concentration, but not in heat-inactivated (HI) serum (**Supplementary Fig. 1a**). A similar number of bacteria was present in serum-survival, HI serum-survival and control (without serum) groups when they were in the same OD (**Supplementary Fig. 1b**). To perform metabolome profiling, six biologically equivalent samples and two technical replicas were prepared for each group, yielding a total of 36 data sets and the abundance of all metabolites was determined and compared. The reproducibility of the GC-MS data is shown in (**Supplementary Fig. 1c)**. A total of 73 metabolites were identified by GC-MS in each of the three groups. Hierarchical clustering for the complete data set is shown in (**Supplementary Fig. 1d).** The statistical significance of differential metabolite abundance in the presence *vs*. the absence of serum is presented as Z-score (**Supplementary Fig. 1e**).

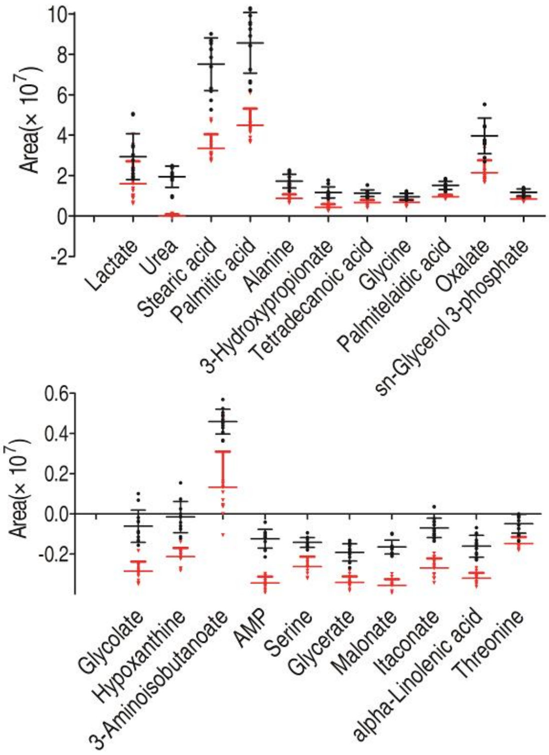

Supplementary Fig. 2 Decreased abundance of metabolites. The data were median centered and inter-quartile range (IQR) scaled per sample in serum-treated samples (red) compared with control (black) from data (**Fig. 1e**) (n = 6). Results are displayed as mean ± SEM, and significant differences are identified (**p < 0.01) as determined by two-tailed Student's t test.

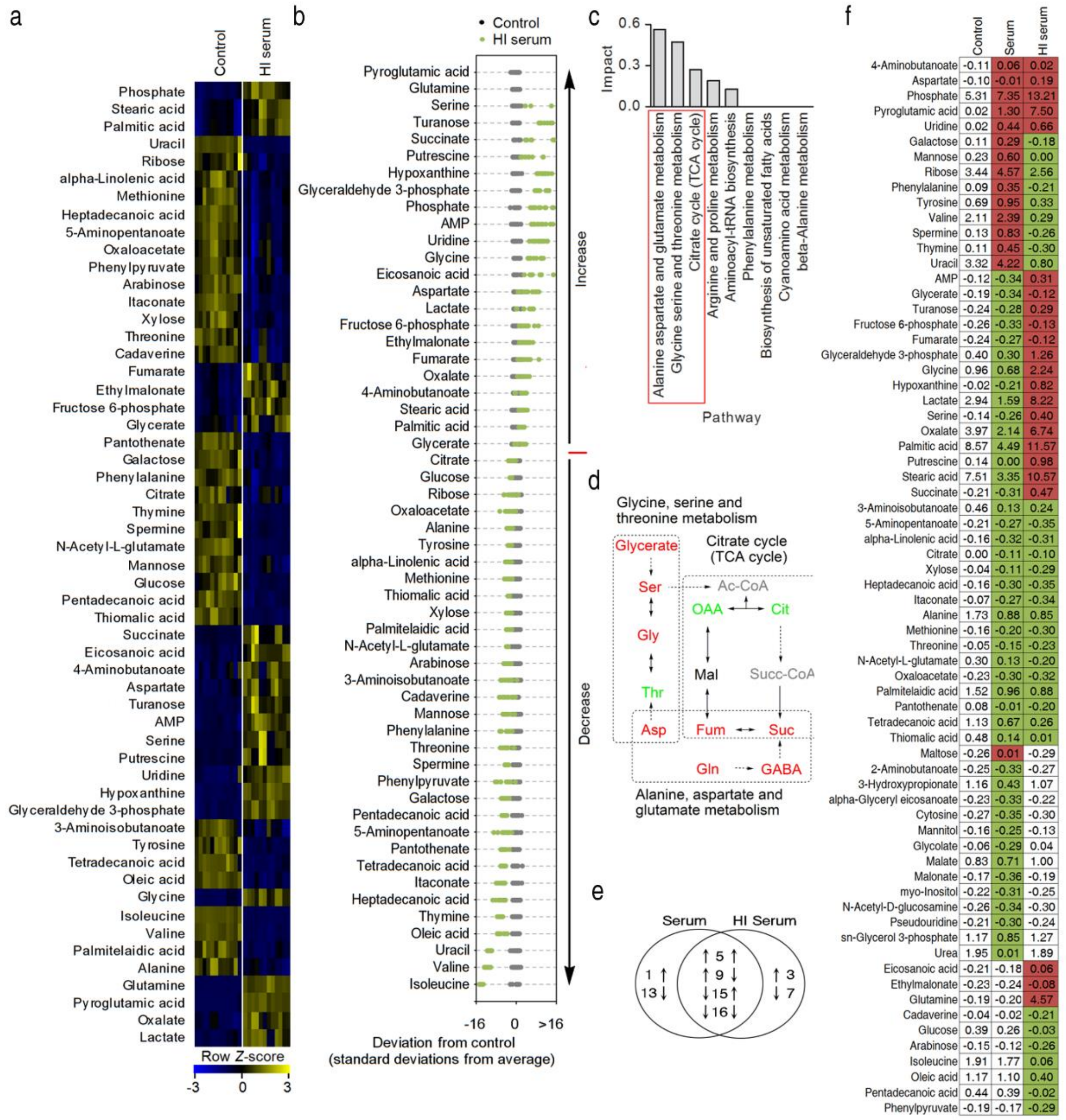

Supplementary Fig. 3 Metabolomic profile for HI serum. a, Heat map showing relative abundance of 55 significantly differential metabolites in *E. coli* K12 in the absence (left) and presence (right) of HI serum as indicated. Heat map scale (blue to yellow; low to high abundance) is shown below data (n = 6). **b,** Z-scores (standard deviation from average) corresponding to data in (**a**). **c,** Pathway enrichment analysis of significantly differential metabolites. Red box highlights putative pathway biomarkers. **d,** Pathway regulation analysis of putative pathway biomarkers. Change in metabolite abundance is indicated as follows: black, no change; red, up-regulation; green, down-regulation; gray, not detected. Thr, threonine; Gly, glycine; Ser, serine; Ac-CoA, acetyl-CoA; Cit, citrate; SuccCoA, succinyl-CoA; Suc, succinate; Fum, fumarate; Mal, malate; OAA, oxaloacetate; Asp, aspartate; Gln, glutamine; GABA, 4-aminobutanoate. **e,** Venn diagram showing the overlapping of differential metabolites between serum and HI serum. Increased and decreased metabolites are indicated in an arrow. **f**, Integrative analysis of these metabolites from data (**e**). Red and green indicate up-regulation and down-regulation of metabolites, respectively. The number shows the normalized abundance of differential metabolites.

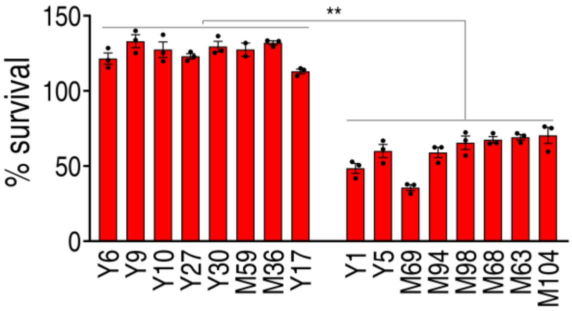

Supplementary Fig. 4 Identification of serum-resistant and –susceptible *E. coli*. Percent survival of sixteen clinically isolated pathogenic *E. coli* strains in the presence of 100 μL serum. Left, serum-resistant strains, right, serum-susceptible strains (*n* = 3). Results are displayed as mean ± SEM, and significant differences are identified (**p < 0.01) as determined by two tailed Student's *t* test.

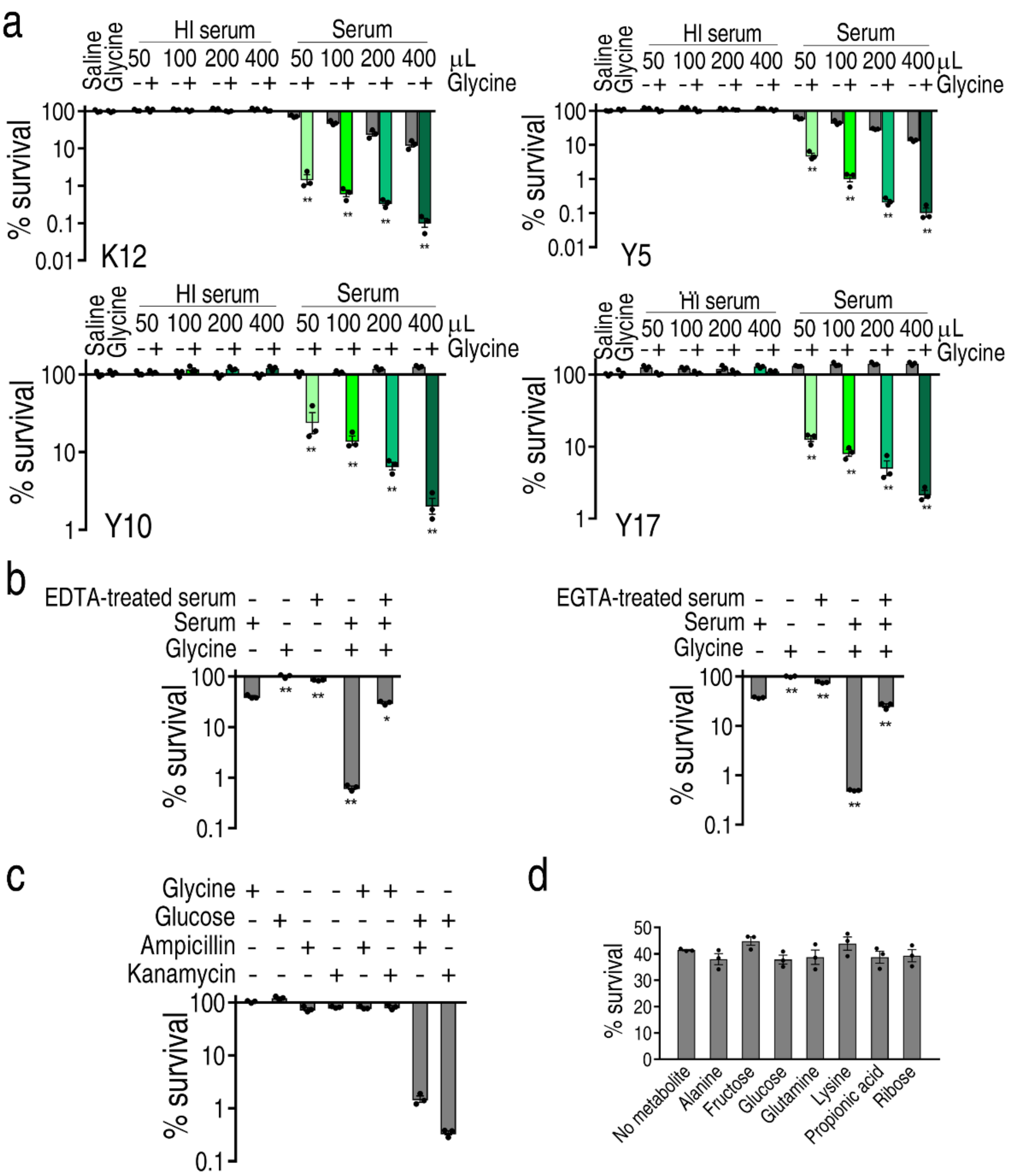

Supplementary Fig. 5 Glycine-enabled killing by serum. a, Percent survival of *E. coli* K12, Y5, Y10 and Y17 in the presence or absence of the indicated volume of serum or HI serum plus 100 mM glycine (n = 3). **b,** Percent survival of *E. coli* K12 incubated in 100 µL serum, or EDTA-, or EGTA-treated serum in the presence or absence of 100 mM glycine (n = 3). **c,** Percent survival of *E. coli* K12 incubated in saline solution and in the indicated metabolites (100 mM glycine or 10 mM glucose) or/and antibiotics (40 µg ampicillin or 2.5 µg gentamicin) for 2 h (n = 3). **d,** Percent survival of *E. coli* K12 in the presence or absence of the indicated metabolites plus 100 µL serum (n = 3). Results (**a-d**) are displayed as mean ± SEM, and significant differences are identified (*p < 0.05; **p < 0.01) as determined by two-tailed Student's *t* test.

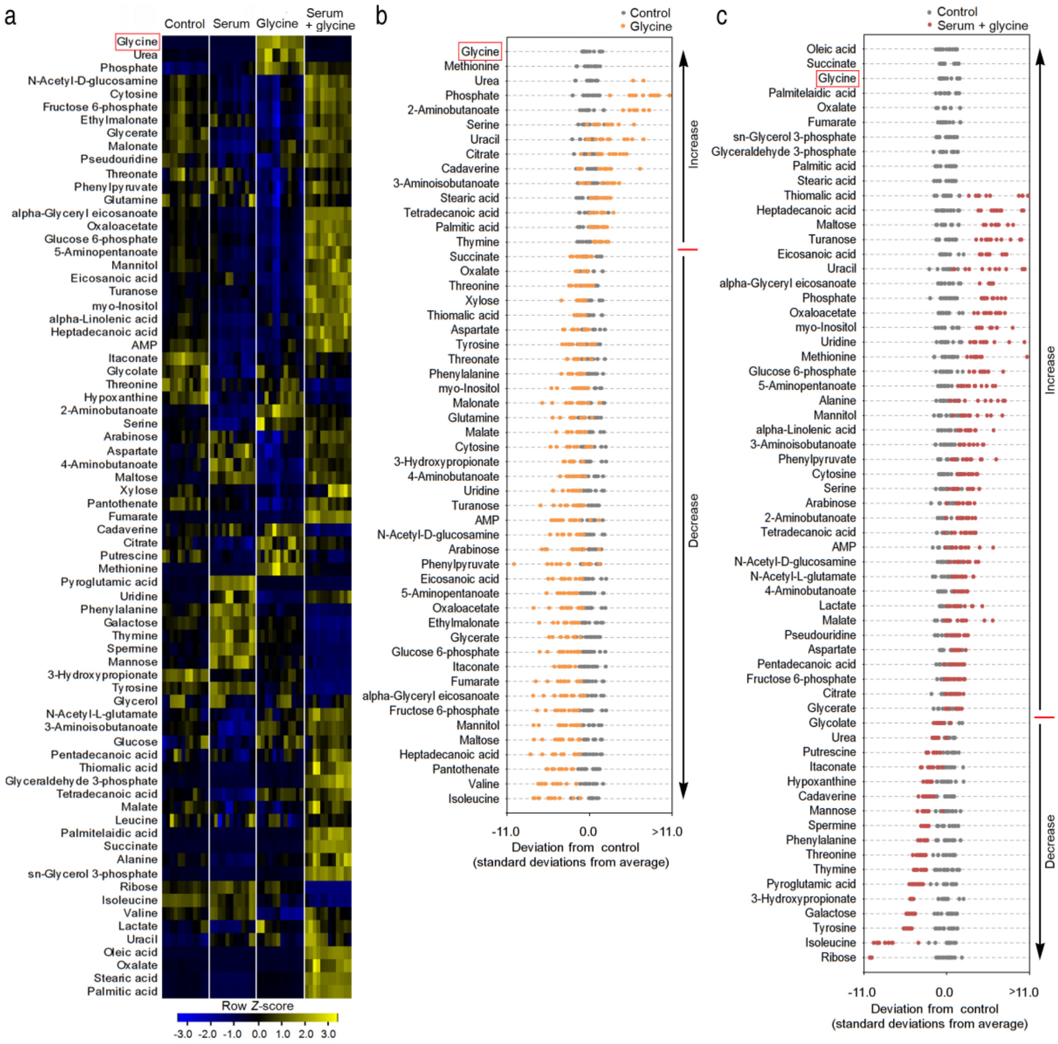

Supplementary Fig. 6 Metabolomic profile for glycine and serum plus glycine. a, Heat map representation of unsupervised hierarchical clustering for metabolites in control, serum, glycine, and serum plus glycine. 73 metabolites in cells were measured across the four groups with 6 independent biological repeats. Yellow and blue indicate an increase and decrease of metabolites relative to the median metabolite level, respectively (see color scale) (n = 6). **b, c**, Z-score plots for the data in treatment groups compared to the mean and standard deviation of the control sample. Further use of Z-scores visualized the change of these metabolites. Significant changes took place in glycine and serum + glycine, relative to the control. Compared with the control, 52 out of the 73 metabolites (71.2%) were significantly differential in glycine, corresponding to false discovery rate (FDR) of 13.0%, in which 14 upregulation and 38 downregulation were detected (**b**). Data showed changes in the Z-scores, which ranged from -10.1 to 622.5 in glycine and from -10.3 to 174.1 in serum + glycine. 63 out of 73 metabolites (86.3%) showed a significant difference in serum + glycine, corresponding to FDR of 4.5%, in which 46 upregulation and 17 downregulation were detected (**c**). Comparison between control and serum is described in **Fig. 1a-e** and **Supplementary Fig. 1c-d**.

Six independent biological replicates with two technical replicates were made for

each group. Unsupervised hierarchical clustering of the high and low abundance metabolites among the four groups is shown in (**Supplementary Fig. 6)**. The critical metabolites were identified by ICA and Kruskal-Wallis' analysis (**Supplementary Figs. 7a** and **7b**). Pathway analysis enriched the same first impactful three pathways in the glycine group and serum plus glycine group (**Supplementary Fig. 7c)**, which is the same as (**Fig. 1c).**

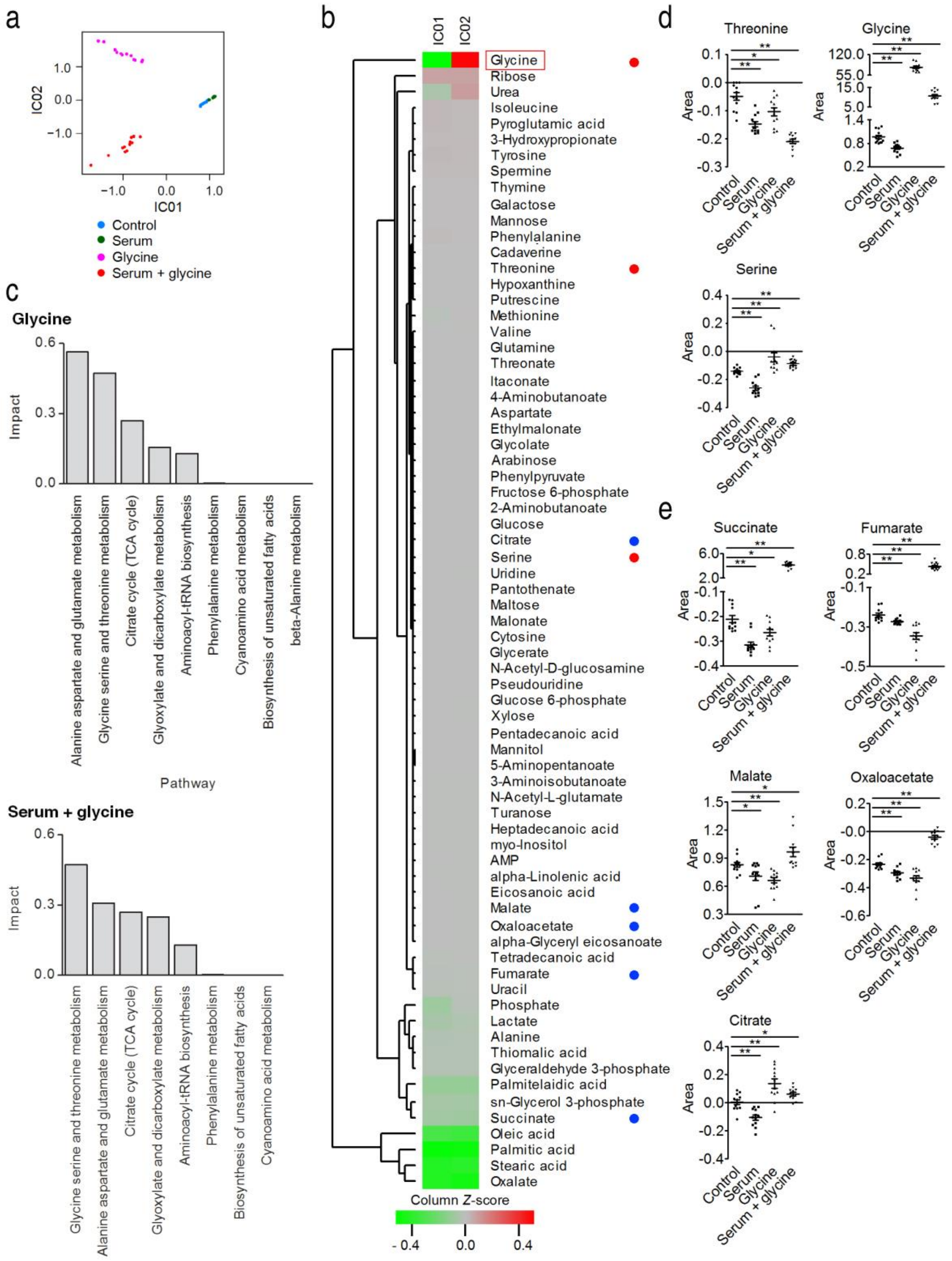

Supplementary Fig. 7 Pathway enrichment analysis and ICA. a, Independent component analysis (ICA) directly captures metabolite variation in four groups, according to the treatments set and the classes of data. Each dot in the plot represents the replicate analysis of cell samples. IC01 and IC02 used in this plot explain 98.34% of the total variance which allows confident interpretation of the variation. Positive IC01 differentiated serum from control and negative IC01 differentiated control from glycine and serum plus glycine. IC02 distinguished glycine from serum plus glycine. **b,** Hierarchical clustering of 71 differential metabolites (multiple comparisons, Kruskal-Wallis, P < 0.05) identified by their weight in different independent components. Red and blue circles indicate the core metabolites in the pathway of

glycine, serine and threonine metabolism and TCA cycle, respectively. Red box highlights the biomarker (*n* = 6). **c,** Pathway enrichment analysis of significantly differential metabolites. **d, e,** Scatter plot of eight core metabolites in the pathway of glycine, serine and threonine metabolism (**d**) and the TCA cycle (**e**) (*n* = 6). Results (**d** and **e**) are displayed as mean ± SEM, and significant differences are identified (* $p < 0.05$; ** $p < 0.01$) as determined by Wilcoxon test.

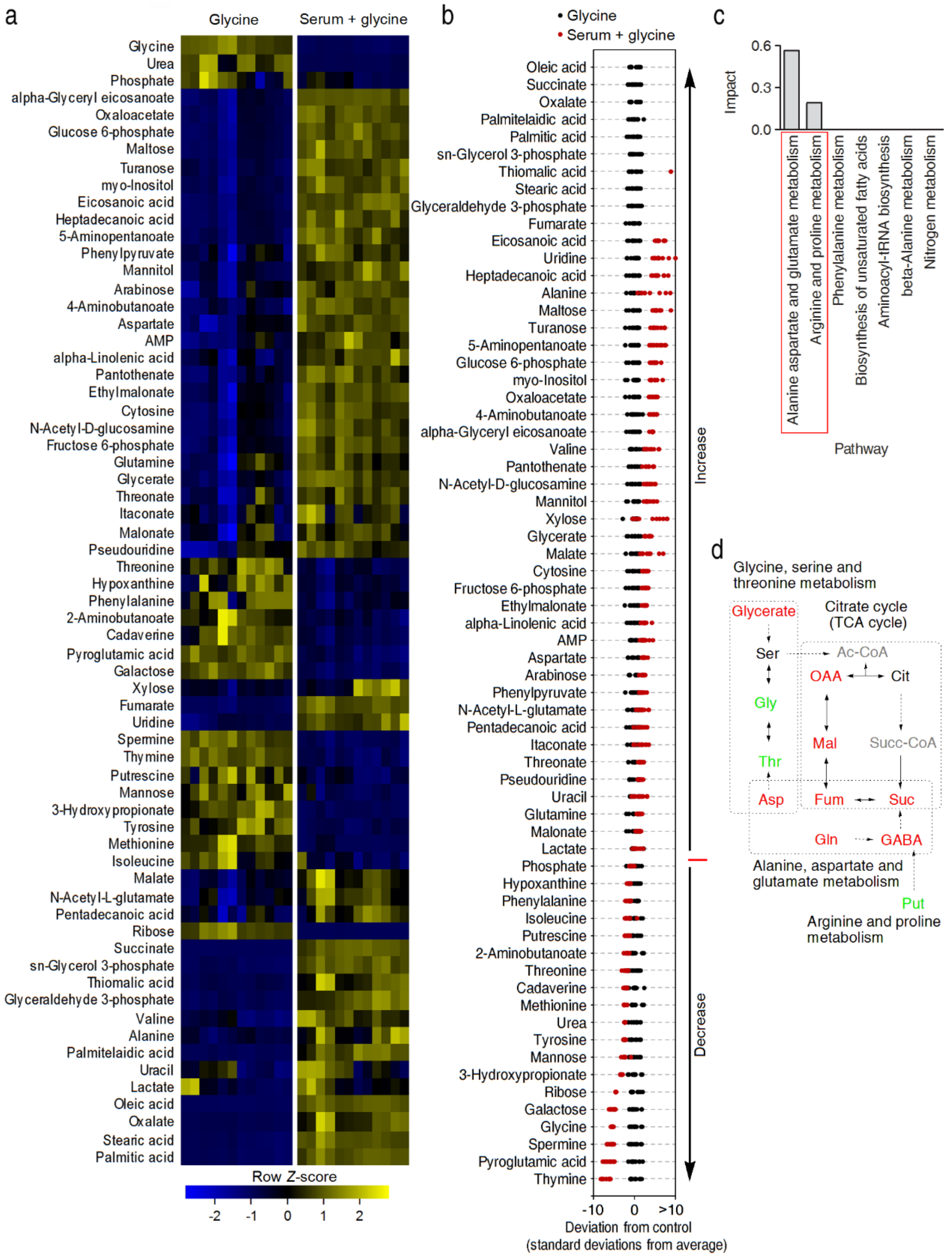

Supplementary Fig. 8 Differential metabolomic profiling. a, Heat map showing the differential metabolites. Yellow and blue indicate an increase and decrease of metabolites relative to the median metabolite level, respectively (see color scale) (n = 6). **b,** Z-score plot for data in serum plus glycine-treated samples compared to the mean and standard deviation of glycine-treated samples with a Z-score range of -8.2 to 118.3. 65 out of the 73 metabolites (89.0%) were significant differential (Wilcoxon P < 0.05), corresponding to false discovery rate of 0.8%. **c**, Pathway enrichment analysis of significantly differential metabolites. Red box highlights putative pathway

biomarkers. **d,** Pathway regulation analysis of putative pathway biomarkers. Change in metabolite abundance is indicated as follows: black, no change; red, up-regulation; green, down-regulation; gray, not detected. Thr, threonine; Gly, glycine; Ser, serine; Ac-CoA, acetyl-CoA; Cit, citrate; SuccCoA, succinyl-CoA; Suc, succinate; Fum, fumarate; Mal, malate; OAA, oxaloacetate; Asp, aspartate; Gln, glutamine; GABA, 4-aminobutanoate; Put, putrescine.

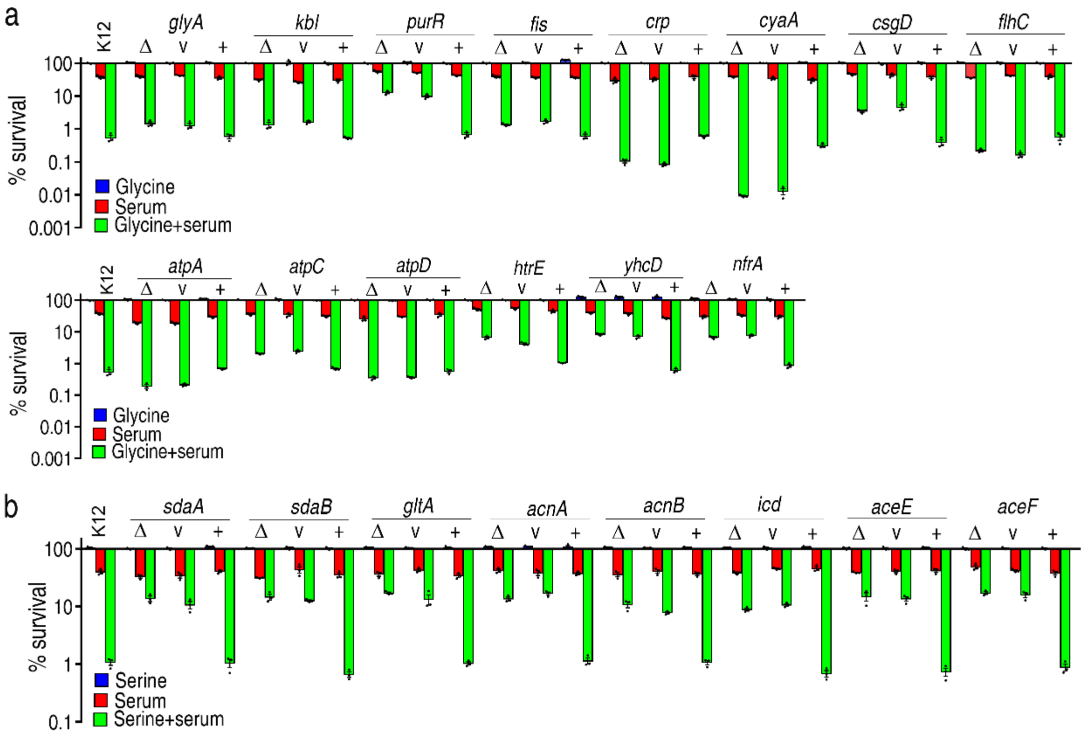

Supplementary Fig. 9 Percent survival of gene-complemented strains. Δ, mutant; V, vector control; +, gene complementation. **a**, 100 µL serum plus 100 mM glycine (*n* = 3). **b**, 100 µL serum plus 50 mM serine (*n* = 3).

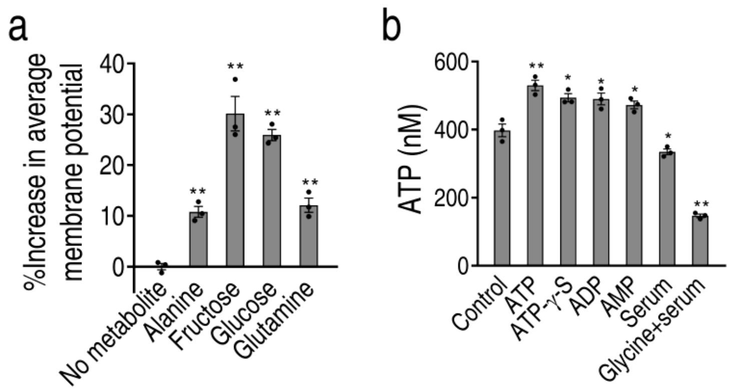

Supplementary Fig. 10 Membrane potential and ATP level. **a**, Percent increased membrane potential in the presence of the indicated metabolites (40 mM alanine, 10 mM fructose, 10 mM glucose, 20 mM glutamine) (*n* = 3). **b,** ATP level of *E. coli* K12 in the presence of the indicated metabolites or/and serum (2 mM ATP, 2 mM ATP-γ-S，2 mM ADP，2 mM AMP；100 μL serum, 100 mM glycine) (*n* = 3). Results are displayed as mean ± SEM, and significant differences are identified (* $p < 0.05$; ** $p < 0.01$) as determined by two-tailed Student's *t* test.

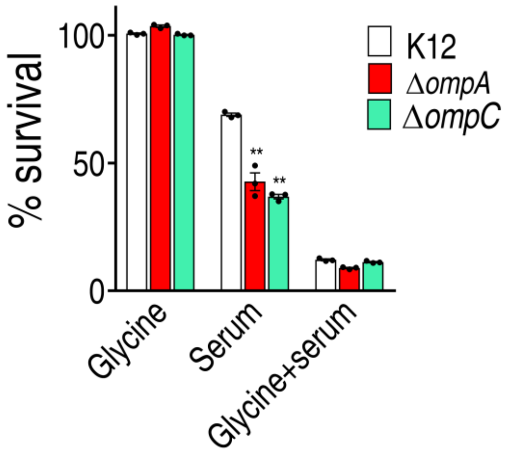

Supplementary Fig. 11 Effect of loss of *ompA*, *ompC* on survival. Percent survival of *ompA*-, *ompC*-deficient mutants in the presence of 100 mM glycine, 100 μL serum or both (*n* = 3)**.** To exclude the possibility that downregulation of outer membrane proteins, including those which are known to interact with complement regulators, e.g. OmpA and OmpC, by exogenous glycine would also increase serum susceptibility, we detected percent survival of *ompA*- and *ompC*-deleted mutants, and found that loss of *ompA* and *ompC* led to lower percent survival in the presence of serum but not in the presence of serum and glycine. Results are displayed as mean ± SEM, and significant differences are identified (** $p < 0.01$) as determined by two-tailed Student’s *t* test.

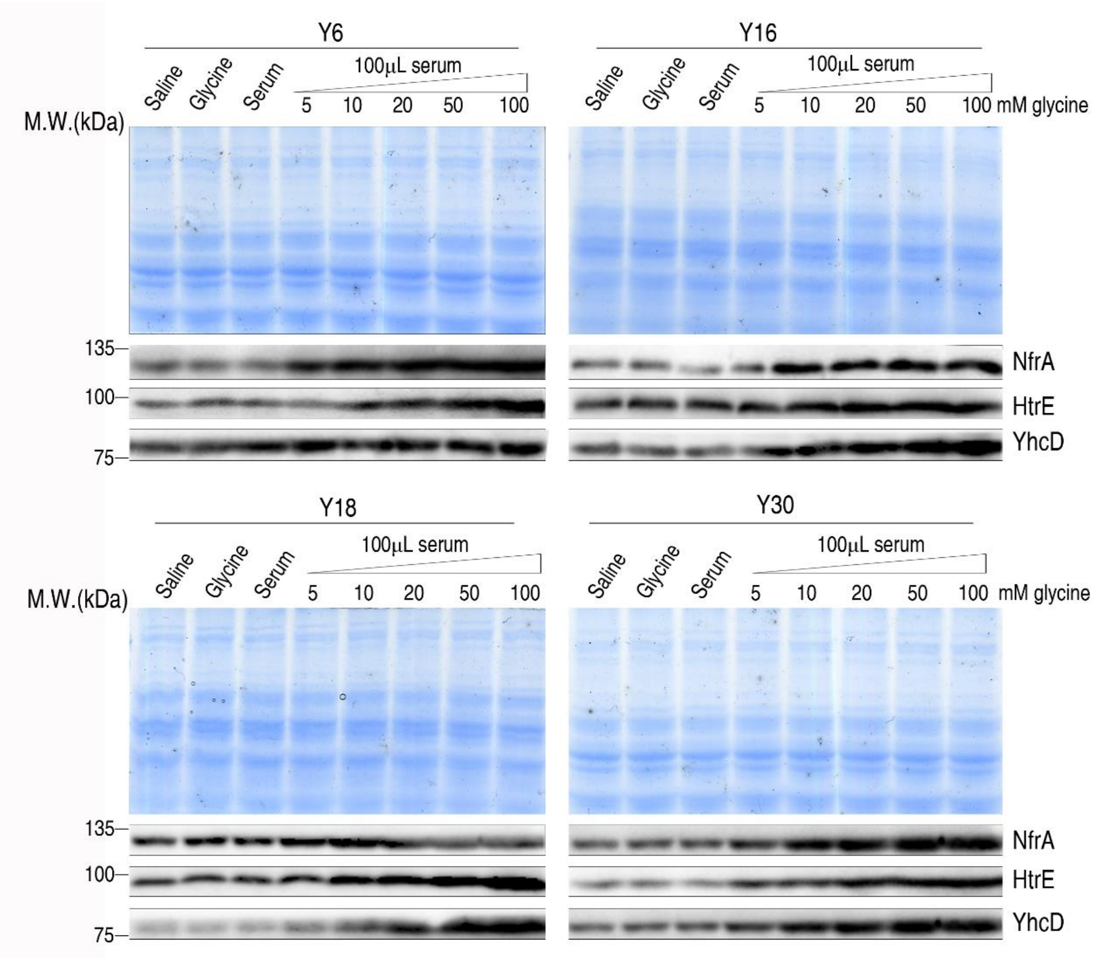

Supplementary Fig. 12 Regulation of NfrA, HtrE and YhcD expression. NfrA, HtrE and YhcD expression induced by increasing concentrations of glycine plus serum in clinically isolated *E. coli* strains Y6, Y16, Y18, and Y30.

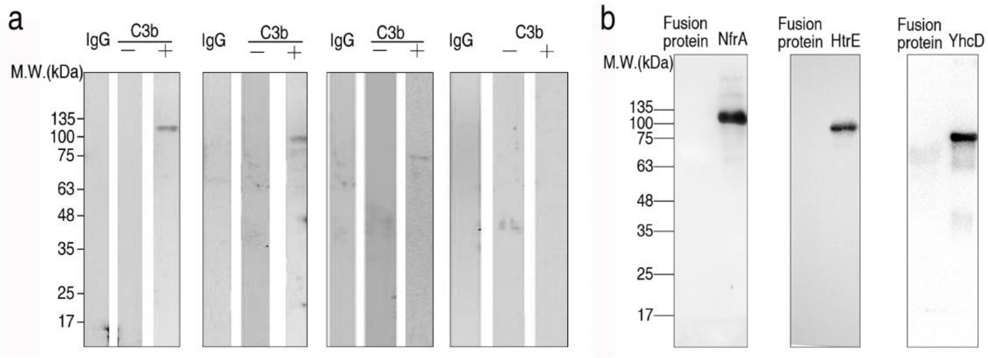

Supplementary Fig. 13 Interaction of C3 with HtrE, NfrA, and YhcD. Far-Western blot and Co-immunoprecipitation (Co-IP) were used for the interaction of C3 with HtrE, NfrA, and YhcD. **a,** Far-Western blot analysis of HtrE, NfrA, and YhcD interacting with complement C3 or IgG. **b,** Co-IP of HtrE, NfrA and YhcD with complement C3. Beads coupled with C3b antibody were incubated with a human serum to capture C3b as a bait protein. The bait protein was reacted with recombinant proteins NfrA, HtrE, and YhcD; blots were probed with anti- HtrE, -NfrA and -YhcD.

a

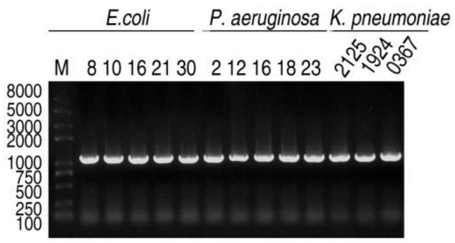

b

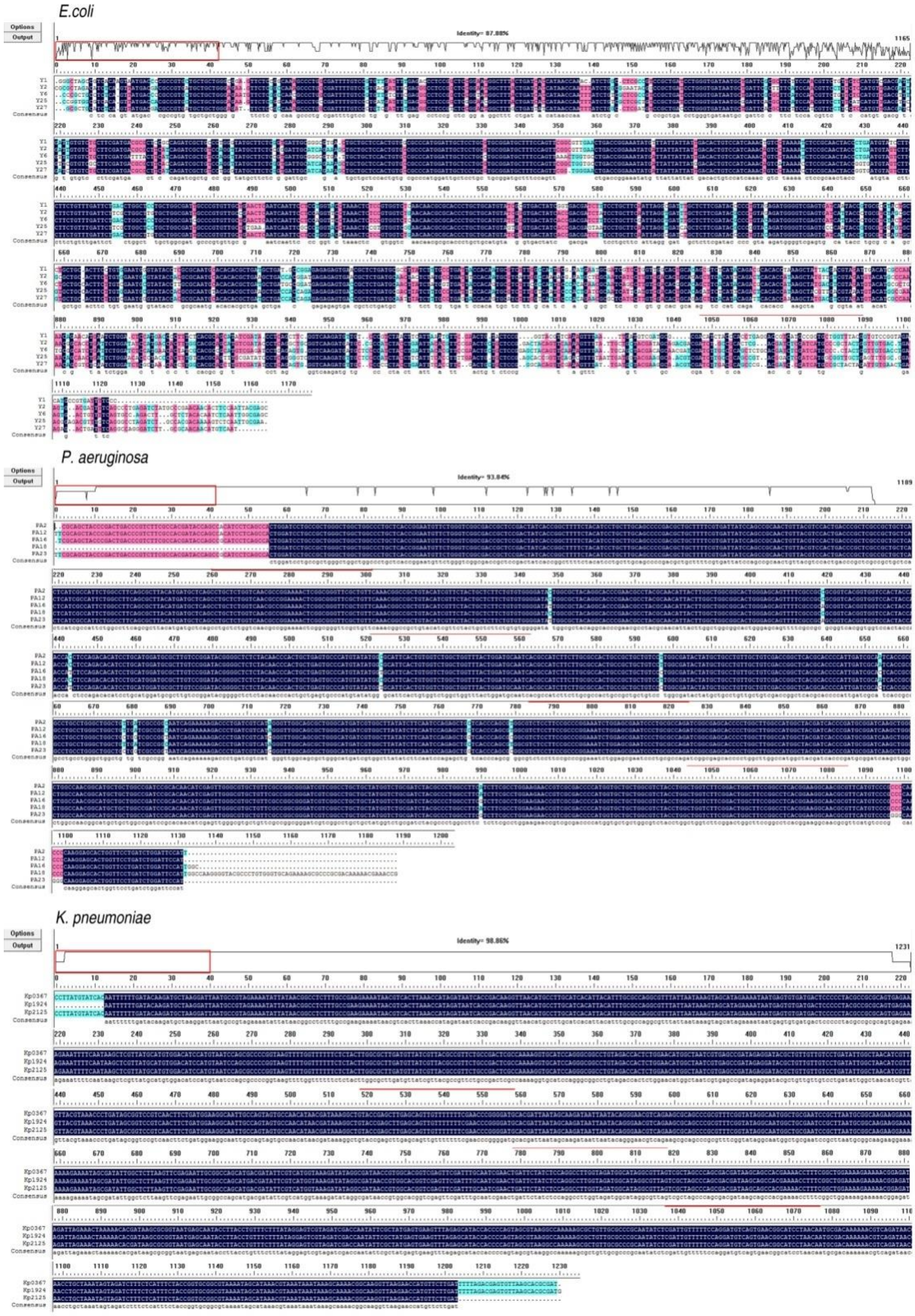

Supplementary Fig. 14 PCR and sequencing of *waal*. PCR (a) and sequencing (b) of *waal* in *E. coli*, *P. aeruginosa*, and *K. pneumonia* strains.

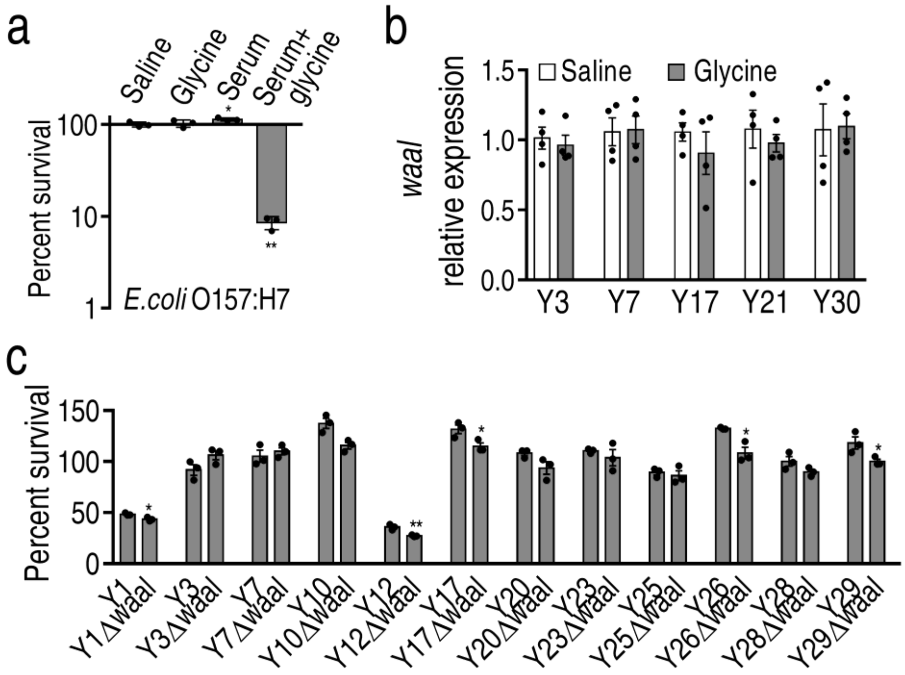

Supplementary Fig. 15 Effect of O-antigens on glycine-mediated serum killing. **a**, Percent survival of *E. coli* O157:H7 with or without 100 μL serum or 100 mM glycine or both (n = 3). **b**, qRT-PCR for detection of *waal* expression in the presence or absence of 100 mM glycine (n = 4). **c**, Percent survival of the indicated *E. coli* strains and their *waal*-deteled mutants in the presence of 100 μL serum (n = 3). Results are displayed as mean ± SEM, and significant differences are identified (*p < 0.05, **p < 0.01) as determined by two-tailed Student's *t* test.

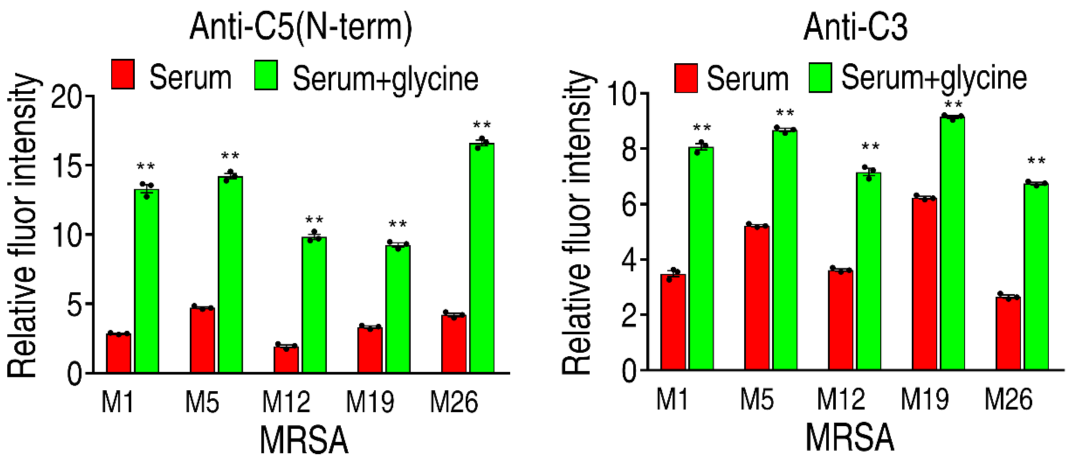

Supplementary Fig. 16 Quantification of C3 and C5 on MRSA membrane. Flow cytometry quantification of C3 and C5 on the membrane surface of the indicated MRSA strains (*n* = 3). Results are displayed as mean ± SEM, and significant differences are identified (*p < 0.05, **p < 0.01) as determined by two-tailed Student's *t* test.

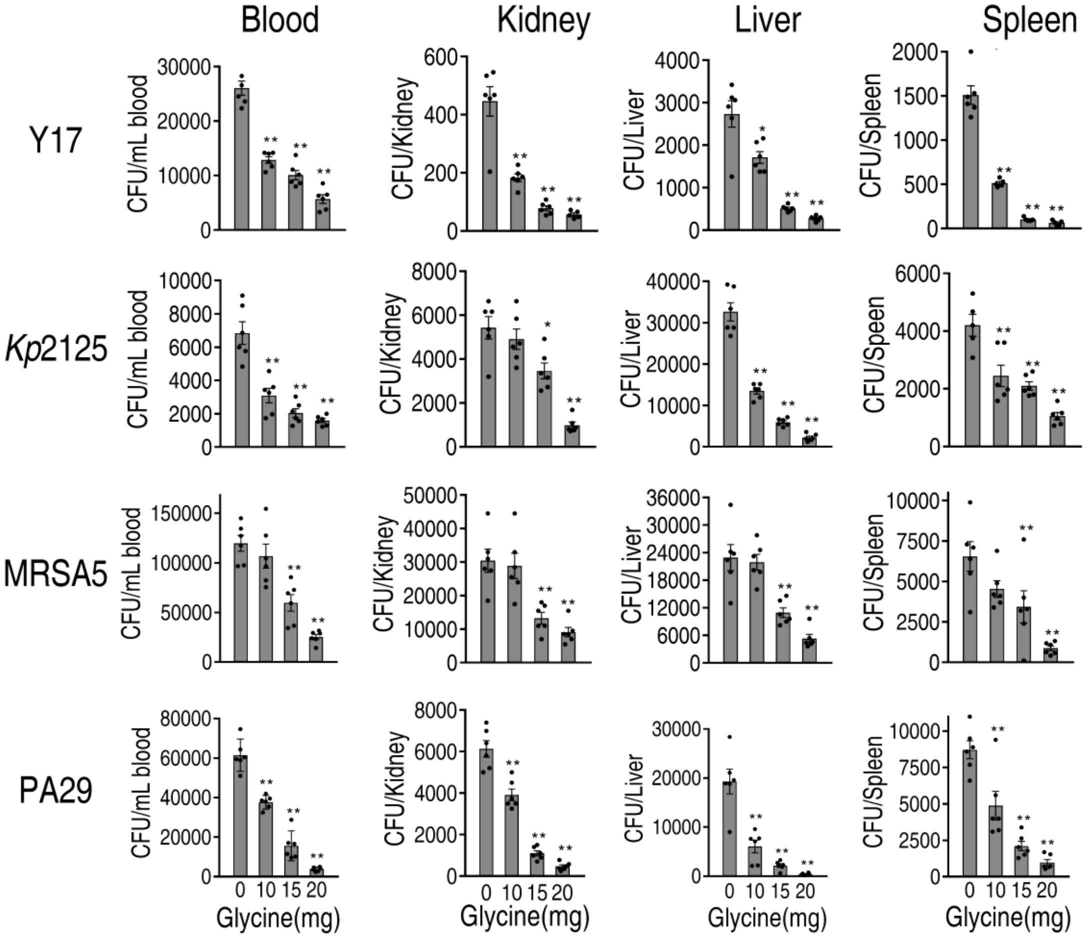

Supplementary Fig. 17 The glycine-enabled killing of clinical strains *in vivo*. The glycine-enabled killing of clinical strains was performed in BALB/c mouse model of bacteremia. Mice were infected by *i. p.* injection (see methods) and treated with glycine as described (see text) (n = 6). Results are displayed as mean ± SEM, and significant differences are identified (*p < 0.05, **p < 0.01) as determined by two-tailed Student's *t* test.

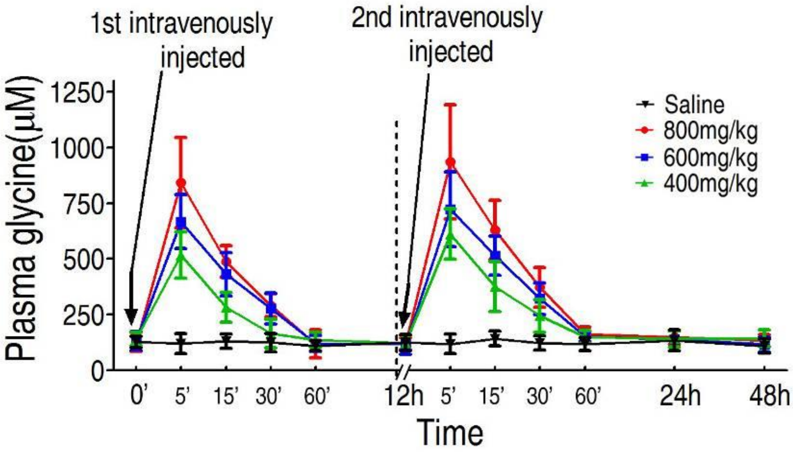

Supplementary Fig. 18 UPLC/MS quantification of plasma glycine. A total of two injections were given, where the second injection was performed 12 h after the first injection (n = 10).

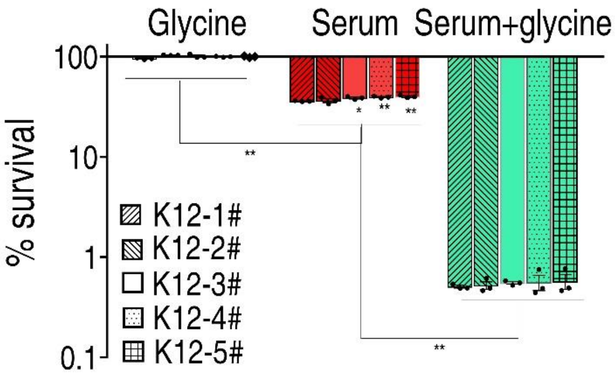

Supplementary Fig. 19 Effect of five round serum killing on survival. Percent survival of *E. coli* K12 recovered from serum killing in the presence of glycine, serum or serum plus glycine (n = 3)**.** Results are displayed as mean ± SEM, and significant differences are identified (*p < 0.05, **p < 0.01) as determined by two-tailed Student's t test.

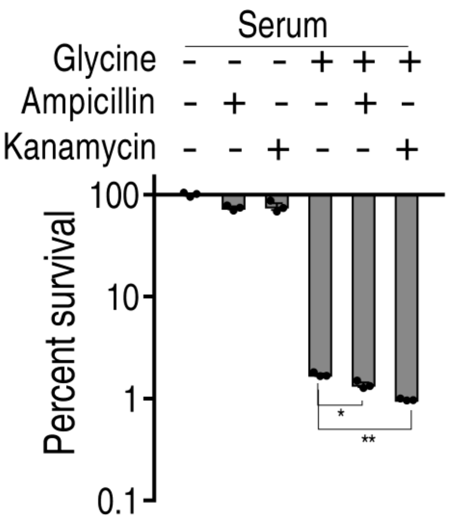

Supplementary Fig. 20 Glycine promotes antibiotics-mediated killing. Percent survival of *E. coli* in M9 and in the presence or absence of the indicated metabolites or/and antibiotics plus serum (*n* = 3)**.** Results are displayed as mean ± SEM, and significant differences are identified (*p < 0.05, **p < 0.01) as determined by two-tailed Student's *t* test. Compared with Supplementary 5c, this result suggests ampicillin and gentamicin-mediated killing is related to environments.

Supplementary Tables

Supplementary Tab. 1 Summary of all compounds which have been detected as labeled by the[U-^{13}C] glycine

RT	Name	Glycine(^{13}C) fragments
8.51	Glycine	1
12.63		1
12.87	Succinate	3
13.82	Fumarate	2
14.45		1
14.05	Serine	1
14.70	Threonine	1
15.99		1
17.27	Malate	3
17.40		2
17.47		2
24.66	Citrate	3
24.98		1
25.59		2

Supplementary Tab. 2 Summary of all compounds which have been detected as labeled by the[U-^{13}C] glycine

RT	Name	Frag	R^2	M0		M1		M2		M3		M4		M5		M6	
				%	SD	%	SD	%	SD	%	SD	%	SD	%	SD	%	SD
12.63	Glycine	248	1	57.6348	4.6702	1.2722	0.1420	41.0930	4.5506								
14.69	Threonine	73	1	51.0989	1.32204	5.48509	0.217	34.8729	1.27231	3.24037	0.30072	5.3027	0.38699				
		219	0.99	71.1429	1.33104	18.5393	1.3153	8.38118	0.75786	1.55078	0.50995	0.38587	0.1849				
14.05	Serine	73	1	56.1061	2.28012	6.11031	0.4121	34.7337	2.36329	3.04987	0.44212						
		204	1	62.7696	3.45678	20.5192	1.5173	8.91253	0.99291	7.7987	1.57336						
24.66	Citrate	273	0.99	64.70363	0.803664	21.11298	1.005435	9.69548	0.578886	3.466182	0.394317	1.021735	0.156035	0.263781	0.131068	0.146062	0.061967
12.87	Succinate	247	1	66.69215	1.191592	19.13368	0.351637	9.482885	0.560201	3.710366	0.211172	0.980925	0.10835				
13.82	Fumarate	245	1	78.81966	2.348887	12.67219	1.362162	6.126606	0.604203	1.89176	0.29757	0.48979	0.103915				
17.27	Malate	233	1	72.07981	2.268233	16.70214	1.651779	8.036514	1.505461	2.613666	0.76606	0.567878	0.586713				

The flux was analyzed in Fig. 3f with supplementary tabs 1 and 2 following as: $^{13}C_2$-glycine tracer experiment was carried out in *E. coli* K12 by GC-MS. Since reciprocal transformation occurs between glycine and serine or threonine, higher M2 labeled serine or threonine was detected. The M2 labeled serine was converted to generate M1 labeled acetyl-CoA via pyruvate dehydrogenase. The M1 labeled acetyl-CoA provides an acetyl-group to citrate, succinate, fumarate, malate, and oxaloacetate to produce M1 label in the initial cycle. In the second cycle, the M1 labeled oxaloacetate and M1 labeled acetyl-CoA generate M2 label. In the following cycles, the M2 labeled oxaloacetate with M1 labeled acetyl-CoA produces M3 label, which formed M4 label with M1 labeled acetyl-CoA. Since an equal amount of M1, M2 and M3 were required for generation of M2, M3, and M4, respectively, the relative flux for that metabolite in the TCA cycle ($\upsilon_{TCA}/\upsilon_{GLY}$) is defined (M2 + M3 + M4) / (M1 + M2 + M3) ratio, where υ_{TCA} refers to the turnover of a particular metabolite pool and υ_{GLY} refers to the flux of glycine carbon atoms to the TCA cycle.

Supplementary Tab. 3 O-antigen distribution of clinically isolated strains

Strains	O-antigen	Strains	O-antigen	Strains	O-antigen
Y1	O55:K59	Y11	O29:K?	Y21	O142:K86
Y2	O124:K72	Y12	O152:K?	Y22	O125:K70
Y3	O119:K69	Y13	O6:K15	Y23	
Y4	O127a:K63	Y14		Y24	O144:K?
Y5		Y15	O119:K69	Y25	O112:K66
Y6	O28:K73	Y16	O25:K19	Y26	O20:K17
Y7	O8:K40	Y17	O126:K71	Y27	O152:K?
Y8	O143:K?	Y18	O9:K9	Y28	O128:K67
Y9	O78:K80	Y19	O29:K?	Y29	O26:K60
Y10	O119:K69	Y20	O136:K78	Y30	O7:K1

Note: ? is marked by the Kit, indicating it is not exactly identified.

Supplementary Tab. 4 Primers for validations of mutants

Gene	Primer sequence (5' to 3')	bp	Gene	Primer sequence (5' to 3')	bp
glyA	F: CTGAATTTTGCAGAAGTGTTAACGC	27	*atpA*	F: CGTTATCATCCGAGCGGGTGATATG	27
	R: TAATCAGCAGATCGTTATGCCCGAG	27		R: GTGTTCTGGACGCTTGCGATCTTAC	27
kbl	F: CCTGAATTTTGCAGAAGTGTTAACG	27	*atpC*	F: AATACGATCACCTGCCGGAGCAGGC	27
	R: TCAGCAGATCGTTATGCCCGAGTTC	27		R: ATATCAGCAGGATCTATGTGAACGC	27
purR	F: TTTACCACTTCCCCTTTTCGTCAAGA	25	*atpD*	F: TATACAACAAAGCTCGTCAGGCCAG	27
	R: GGAGAATTCGGTGCCAAACCGCTT	25		R: GTTACCTGGATTTTCTCGACCAGAC	27
purD	F: GTGAAAGGTTCCTCGATGGCTTCTGA	25	*sdaA*	F: TTCATGTGAATAGTTAAGCCAGTCG	27
	R: GTCGAGCGCAAGCCTTTATATAAG	25		R: ACGCCTGACGCAACAGTGGAAGTGT	27
nfrA	F:CGCGTCCTGACAATTCAACGCGAATTAC	30	*aceE*	F: GCAACTAAACGTAGAACCTGTCTTATTG	30
	R:GACTATCTCGGTTTTGTGGTTGCCAGAT	30		R: TCAACTTTGTCGCCCACTTTGACCAGG	29
htrE	F: AGTGCATTTTTACGCCATTAATGAC	27	*aceF*	F: TGGCGAAATCGATAAGAAAGTGGTTGC	29
	R: ATATCCGTTGCAAATACTGAGGGTG	27		R: CAAACGGCAACCGATTTGTCTATTCGC	29
yhcD	F: TACGGATACAAAAGTCGAGTTTTAC	27	*gltA*	F: AGCGGCGAGCCAAATAAAAAACGGG	27
	R: CCATAGTCGATATTTTGTATGCTAC	27		R: AATCAACCCGCCATATGAACGGCGG	27
csgD	F: TTACTACACACAGCAGTGCAACATC	27	*acnA*	F: ATTTGGGTTGTTATCAAATCGTTACGCG	30
	R: AGTTAGCAATCCCGGGACTTCTACC	27		R: CAAAGCCGAGAAAATGGGCCATTTGCTG	30
flhC	F: ACGATCTCCAGCAAATTCATACCGG	27	*acnB*	F: CCGTTGCGGTACTGGGCATTTACCCTAC	30
	R: GACAGGATGTTCAGTCGTCAGGCGT	27		R: GAAAAACAGCGGAACGGCGATGGTTTAG	30
crp	F: TGATAGCCCCTTCCCAGGTAGCGGG	27	*icd*	F: TCTAAAGCATCCGTATCGCAGGACG	27
	R: TGTCGAAGTGCATAGTTGATATCGG	27		R: TTTTGATAACCGTTAGCAGCCTAAC	27
fis	F: CGCACATTCAACGCCATTGAGGATG	27	*cyaA*	F: CGGTTACCTTTGACATACGAAATATCCCG	31
	R: ACATCCTGTTCTCATGGTCACTCCC	27		R:CTGGGATTTGCTGGAACAGGCGGCGACGC	31

Supplementary Tab. 5 Bacterial strains used in the study

Strain	Original	Strain	Original	Strain	Original
E.coli K12[1]	KEIO collection	Δ*sdhD*	KEIO collection	Δ*sdaA*	KEIO collection
Δ*nfrA*	KEIO collection	Δ*frdA*	KEIO collection	Δ*sdaB*	KEIO collection
Δ*htrE*	KEIO collection	Δ*frdB*	KEIO collection	Δ*tdcG*	KEIO collection
Δ*yhcD*	KEIO collection	Δ*frdB*	KEIO collection	Δ*trpA*	KEIO collection
Δ*purR*	KEIO collection	Δ*frdC*	KEIO collection	Δ*trpB*	KEIO collection
Δ*fis*	KEIO collection	Δ*frdD*	KEIO collection	Δ*tynA*	KEIO collection
Δ*crp*	KEIO collection	Δ*mdh*	KEIO collection	Δ*aecE*	KEIO collection
Δ*cyaA*	KEIO collection	Δ*mqo*	KEIO collection	Δ*aecF*	KEIO collection
Δ*flhC*	KEIO collection	Δ*purL*	KEIO collection	Δ*gltA*	KEIO collection
Δ*csgD*	KEIO collection	Δ*purK*	KEIO collection	Δ*acnA*	KEIO collection
Δ*atpA*	KEIO collection	Δ*purE*	KEIO collection	Δ*acnB*	KEIO collection
Δ*atpC*	KEIO collection	Δ*purC*	KEIO collection	Δ*ybhJ*	KEIO collection
Δ*atpD*	KEIO collection	Δ*purH*	KEIO collection	Δ*icd*	KEIO collection
Δ*glyA*	KEIO collection	Δ*guaA*	KEIO collection	Δ*sucA*	KEIO collection
Δ*kbl*	KEIO collection	Δ*guaB*	KEIO collection	Δ*sucB*	KEIO collection
Δ*purD*	KEIO collection	Δ*guaC*	KEIO collection	Δ*sucC*	KEIO collection
Δ*ltaE*	KEIO collection	Δ*ndk*	KEIO collection	Δ*sucD*	KEIO collection
Δ*ilvA*	KEIO collection	Δ*mazG*	KEIO collection	Δ*sdhA*	KEIO collection
Δ*tdcB*	KEIO collection	Δ*gsk*	KEIO collection	Δ*sdhB*	KEIO collection
Δ*pepA*	KEIO collection	Δ*ushA*	KEIO collection	Δ*sdhC*	KEIO collection
Δ*pepB*	KEIO collection	Δ*yibR*	KEIO collection	Δ*pykA*	KEIO collection
Δ*pepN*	KEIO collection	Δ*surE*	KEIO collection	Δ*pykF*	KEIO collection
Δ*gcvP*	KEIO collection	Δ*yjjG*	KEIO collection	Δ*gshB*	KEIO collection
Δ*tdh*	KEIO collection	Y3Δ*glyA*	This study	Y17Δ*waal*	This study

Δ*nfrA*Δ*yhcD*	This study	Y21Δ*glyA*	This study	Y20Δ*waal*	This study
Δ*htrE*Δ*yhcD*	This study	Y1Δ*waal*	This study	Y23Δ*waal*	This study
ΔyhcDΔ*htrE*	This study	Y3Δ*waal*	This study	Y25Δ*waal*	This study
Δ*nfrA*Δ*yhcDΔhtrE*	This study	Y7Δ*waal*	This study	Y26Δ*waal*	This study
ΔhtrEΔyhcDΔ*nfrA*	This study	Y10Δ*waal*	This study	Y28Δ*waal*	This study
ΔyhcDΔhtrEΔ*nfrA*	This study	Y12Δ*waal*	This study	Y29Δ*waal*	This study
Vibro. alginolyticus			The collections of our laboratory[2]		
Vibro. parahaemolyticus			The collections of our laboratory		
E.coli O157:H7*			The collections of our laboratory[3]		
30 strains of multidrug-resistant *Pseudomonas aeruginosa*		The collections of our laboratory, which were isolated from patients			
30 strains of methicillin-resistant *Staphylococcus aureus* (MRSA)		The collections of our laboratory, which were isolated from patients			
30 strains of multidrug-resistant *Klebsiella pneumonia*		The collections of our laboratory, which were isolated from patients			
30 strains of multidrug-resistant *E.coli*		The collections of our laboratory, which were isolated from patients			

[1] Baba T, *et al*. Construction of *Escherichia coli* K-12 in-frame, single-gene knockout mutants: the Keio collection. *Mol Syst Biol.* **2**, 2006.0008 (2006).
[2] Xiong XP, Wang C, Ye MZ, Yang TC, Peng XX, Li H. Differentially expressed outer membrane proteins of *Vibrio alginolyticus* in response to six types of antibiotics. Mar Biotechnol, 2010, 12:686-695
[3] Li H, Xiong XP, Peng B, Xu CX, Ye MZ, Yang TC, Wang SY, Peng XX. Identification of broad cross-protective immunogens using heterogeneous antiserum-based immunoproteomic approach. J Proteome Res., 2009, 8, 4342–4349

*, The bacterium is with adhesive fimbriae and a cell wall that consists of an outer membrane containing LPS,

Supplementary Tab. 6 Primers for construction of mutants

**Primers for amplification of kanamycin cassette（5'-3'）**	
Y3-*glyA*-KOF	CAGCAAATCACCGTTTCGCTTATGCGTAAACCGGGTAACGTGCG CAGATGATTCCGGGGATCCGTCGACC
Y3-*glyA*-KOR	TTTATTGTTAGCTGAGTCAGGAGATGCGGATGTTAAAGCGTGAAA TGAACTGTAGGCTGGAGCTGCTTCG
Y21-*glyA*-KOF	GGACCGCCTATAAAGGCCAAAAATTTTATTGTTAGCTGAGTCAGG AGATGATTCCGGGGATCCGTCGACC
Y21-*glyA*-KOR	ACGAGCACATTGTCAGCAAATCACCGTTTCGCTTATGCGTAAACC GGGTATGTAGGCTGGAGCTGCTTCG
ECO-*yhcD*-KOF	AATAAATATGTCACTGACTTAAAATAACTTTGCCTGGAGCGACAA GGATGATTCCGGGGATCCGTCGACC
ECO-*yhcD*-KOR	GTTCAGCAGCAGGCATCCTGTTATTATCCGTTTCATTGGCACTCT CCTGT TGTAGGCTGGAGCTGCTTCG
ECO-*htrE*-KOF	TGCCAGGCTGTAATCAGGCAAGGATATAATTCCGCAGGAAGCAT AGCGTGATTCCGGGGATCCGTCGACC
ECO-*htrE*-KOR	GCGTTGTTTTTATCATTTGACGTCCCTTGTAGTTACTGAATCTGAC ACCG TGTAGGCTGGAGCTGCTTCG
ECO-*nfrA*-KOF	CAAAGCAGGTTTAAACACAGAACAGGTTGCGCAACTGGAGTCC GAAAATG ATTCCGGGGATCCGTCGACC
ECO-*nfrA*-KOR	AGTGTCAGCAATACGAAAATGAACTTACGCATTTACCAGTGCAC TCCAAT TGTAGGCTGGAGCTGCTTCG
waal-KOF	ACAGTCAAGCAGTTTTGGAAAAGTTATCATCATTATAAAGGTAAAAC ATG ATTCCGGGGATCCGTCGACC
waal-KOR	AGTTTTAACTCACTTCTTAAACTTGTTTATTCTTAATTAATTGTATTGT T TGTAGGCTGGAGCTGCTTCG
**Test primers**	
Y3-*glyA*-F	GCCTCCGGAGATTGCAATATATT
Y3-*glyA*-R	GATTCTTTGTAGACCTGTTATCGCA
Y21-*glyA*-F	CCGGTAGACCTGTTATCGCAC
Y21-*glyA*-R	ACCGGAGATTGCAATATATTGAA
ECO-*yhcD*-F	AAAGATGACAAGAGCAGCAGAG
ECO-*yhcD*-R	GCTTATTCGCACCTTCCCT
ECO-*htrE*-F	GCACCTCACTGCCTAAAGACA
ECO-*htrE*-R	CTCGTCTGTGCTCCAGCGTA
ECO-*nfrA*-F	ATCAGTGGCTCACGGAACAG
ECO-*nfrA*-R	GTCGTCAATTTCCGCGCT
waal-F	AGAACGGGCGAAACGACT
waal-R	ACCGGGTTACCGCAAGAT

Supplementary Tab. 7 Primers for gene complementation

Gene	Primer sequence (5' to 3')	bp
glyA	F: CCCAAGCTTATGTTAAAGCGTGAAATGAACATTG	36
	R: CGCGGATCCTTATGCGTAAACCGGGTAACGTG	34
kbl	F: CCCAAGCTTATGCGTGGAGAATTTTATCAGCAG	35
	R: CGCGGATCCTCAGGCGATAACGCCCAGTTGTTTA	36
nfrA	F: GACAGCTTATCATCGATAAGCTTATGAAGGAGAATAACCTTAATCG	48
	R: GATGCGTCCGGCGTAGAGGATCCTTACCAGTGCACTCCAATGGTGAG	49
htrE	F: CCCAAGCTTGTGACTATAGAATATACTAAAAATT	36
	R: CGCGGATCCTTACTGAATCTGACACCGAATTCCA	36
yhcD	F: GACAGCTTATCATCGATAAGCTTATGTTAAAAAAAACGTTACTGGC	48
	R: GATGCGTCCGGCGTAGAGGATCCTCATTGGCACTCTCCTGTTGC	46
csgD	F: GACAGCTTATCATCGATAAGCTTATGTTTAATGAAGTCCATAGTATTC	50
	R: GATGCGTCCGGCGTAGAGGATCCTTATCGCCTGAGGTTATCGTTTG	48
flhC	F: CCCAAGCTTATGAGTGAAAAAAGCATTGTT	32
	R: CGCGGATCCTTAAACAGCCTGTACTCTCTG	32
crp	F: CCCAAGCTTATGGTGCTTGGCAAACCGCAAAC	34
	R: CGCGGATCCTTAACGAGTGCCGTAAACGACGATG	36
fis	F: CCCAAGCTTATGTTCGAACAACGCGTAAATTC	34
	R: CGCGGATCCTTAGTTCATGCCGTATTTTTTC	33
atpA	F: CCCAAGCTTATGCAACTGAATTCCACCGAAATCA	36
	R: CGCGGATCCTTACCAGGATTGGGTTGCTTTG	33
atpC	F: CCCAAGCTTATGGCAATGACTTACCACCTG	32
	R: CGCGGATCCTTACATCGCTTTTTTGGTCAAC	33
atpD	F: CCCAAGCTTATGGCTACTGGAAAGATTGTC	32
	R: CGCGGATCCTTAAAGTTTTTTGGCTTTTTCC	33
sdaA	F: CCCAAGCTTGTGATTAGTCTATTCGACATG	32
	R: CGCGGATCCTTAGTCACACTGGACTTTGATTG	34
gltA	F: CCCAAGCTTATGGCTGATACAAAAGCAAAAC	33
	R: CGCGGATCCTTAACGCTTGATATCGCTTTTAAAG	36
acnA	F: GACAGCTTATCATCGATAAGCTTATGTCGTCAACCCTACGAGAAGC	48
	R: GATGCGTCCGGCGTAGAGGATCCTTACTTCAACATATTACGAATGAC	49
acnB	F: CCCAAGCTTGTGCTAGAAGAATACCGTAAGCACG	36
	R: CGCGGATCCTTAAACCGCAGTCTGGAAAATCACC	36
icd	F: CCCAAGCTTATGGAAAGTAAAGTAGTTGTTC	33
	R: CGCGGATCCTTACATGTTTTCGATGATCGCG	33
cyaA	F: GACAGCTTATCATCGATAAGCTTTTGTACCTCTATATTGAGACTC	47
	R: GATGCGTCCGGCGTAGAGGATCCTCACGAAAAATATTGCTGTAATAGC	50
purR	F: CCCAAGCTTATGGCAACAATAAAAGATGTAG	33
	R: CGCGGATCCTTAACGACGATAGTCGCGGAACG	34
aceE	F: GACAGCTTATCATCGATAAGCTTATGTCAGAACGTTTCCCAAATG	47
	R: GATGCGTCCGGCGTAGAGGATCCTTACGCCAGACGCGGGTTAACT	47

aceF	F: GACAGCTTATCATCGATAAGCTTATGGCTATCGAAATCAAAGTACCG	49
	R: GATGCGTCCGGCGTAGAGGATCCTTACATCACCAGACGGCGAATG	47
purD	F: CGCGGATCCATGAAAGTATTAGTGATTGG	29
	R: CCCAAGCTTTTAGTTCTGCTCGCGTTCGAT	30

Supplementary Tab. 8 Primers for qRT-PCR

Gene	Primer	Primer sequence	Product size(bp)
16S rRNA	Forward	5’-ACTGAGACACGGTCCAGACTCCTAC-3’	146
	Reverse	5’-TTAACGTTCACACCTTCCTCCCTAC-3’	
glyA	Forward	5’-AGCCCGTTTGTGACCTCC-3’	91
	Reverse	5’-AGCCAGCCAGTTCTTTCG-3’	
kbl	Forward	5’-TGAATACTGCGATGTGATGGG-3’	146
	Reverse	5’-TGGAGAACAGGTACGGACGAG-3’	
purD	Forward	5’-CAAACAGGGCAATCCGAAGG-3’	191
	Reverse	5’-ATCCACCCGCAGCCATCA-3’	
aceE	Forward	5’-ACCTCTGGACGCACCACCCT-3’	181
	Reverse	5’-AGTACACGTTCTCCTGCTTCTCA-3’	
aceF	Forward	5’-CAGGGCGGCTGCTTCACTA-3’	141
	Reverse	5’-CGCAAACTCTTTCCCATTCCA-3’	
gltA	Forward	5’-TGAGCTGGGTATGAATGACGA-3’	127
	Reverse	5’-CAGTATGATGCCGGAGTAGAAG-3’	
sdaA	Forward	5’-CGGAAATTGGCATGGAAC-3’	111
	Reverse	5’-CGTTAATCGCCTTCACAGAG-3’	
sdaB	Forward	5’-AGCAAAGAAGAGCTGGAACA-3’	85
	Reverse	5’-CGCCTTCGGTGGAAATAC-3’	
tdcG	Forward	5’-CGCCATGAGAACGGAATG-3’	80
	Reverse	5’-CCGCCGACAGAGTAATAGG-3’	
acnA	Forward	5’- ATTGCCCGTGCGGTAGAA-3’	189
	Reverse	5’- CACTGGTGCTGGTGTTGC-3’	
acnB	Forward	5’-TCCCTGTGCATGGGCAACC-3’	194
	Reverse	5’-CCCACCTTCGCCATAAACTGC-3’	
ybhJ	Forward	5’-GTGTTTATCAGGGCTTTGTG-3’	156
	Reverse	5’-TCAGTTCGTCGGTGGTGG-3’	
icd	Forward	5’-AAACGCCGAGGATATTTACGC-3’	126
	Reverse	5’-ATGCCGCAGTGCTCAGGGA-3’	
sucA	Forward	5’-ACCTACTGCGGAACCATCGG-3’	119
	Reverse	5’-TTCTCCTGCGGGCTAAACG-3’	
sucB	Forward	5’-TACGCCGATCATCAACCC-3’	169
	Reverse	5’-CGTTACCAGGAAGCCCAC-3’	
sucC	Forward	5’-GCCGAACAGTGGCTGGGTA-3’	89
	Reverse	5’-GTCGCTCCCTCCACCAGAAT-3’	
sucD	Forward	5’-TGTGGGTTACATCGCTGGTG-3’	87
	Reverse	5’-CAGGCTGCGAACGGTTTT-3’	
tdcB	Forward	5’-ACCCGACCATTCGTGTTAT-3’	143
	Reverse	5’-GGTGCGTGGTTATTTCTCC-3’	
ilvA	Forward	5’-GCGTATTTGTGCCAGTCGG-3’	98

	Reverse	5’-CGCTTCTACGGCGATCACTT-3’	
tdh	Forward	5’-AGGTATTTACGGTCGTGAGATG-3’	132
	Reverse	5’-ATAGCGTCAAAGCCCTTCT-3’	
ltaE	Forward	5’-AATGACAGGTGGCGGGATG-3’	107
	Reverse	5’-AGGCAGCGTTGTCGTGGTC-3’	
gshB	Forward	5’-TGACGGAAAGTGACTGGAAA-3’	131
	Reverse	5’-AATACAGGTTGGGCTGGTG-3’	
gcvP	Forward	5’-GGTGACTTATCCTTCTACCCACG-3’	86
	Reverse	5’-AAACCTGACCGCCGAACTG-3’	
pepA	Forward	5’-TGGGTGACGAGTATCAGGAA-3’	111
	Reverse	5’-GGGTAAAGCGTGACAGGAA-3’	
pepB	Forward	5’-TCCTCTGCTGTGCGGATAA-3’	110
	Reverse	5’-CCCTTCGGCATCAGTGTTC-3’	
pepN	Forward	5’-TGAGCAACCCGAACCGTAT-3’	91
	Reverse	5’-AGGTAACCGCTGCCATCTT-3’	
cycA	Forward	5’-CGTGGTGATGTTGTATGTGAA-3’	142
	Reverse	5’-ATGTAGATGAGGACGCTGTTT-3’	
crp	Forward	5’-TTCTGATGCGTTTGTCTGC-3’	141
	Reverse	5’-GAGTCATAGCGTCTGGTTGTT-3’	
nfrA	Forward	5’-CAACATCGTGCGGTAGCCT-3’	121
	Reverse	5’-TATTGGCAGCAGCAAGCAG-3’	
htrE	Forward	5’-GTCTTATACGACGGGCGAAAC-3’	94
	Reverse	5’-GCTGGCTAAAGTCGGAGGC-3’	
yhcD	Forward	5’-CATCGCAGGATACGAAAGA-3’	90
	Reverse	5’-TGGTTGTTAGCCCAAGTGA-3’	
flhC	Forward	5’-CGTGCCTGGACATTGGTGC-3’	99
	Reverse	5’-AGGCTGGTGAGCGTGGGTA-3’	
csgD	Forward	5’-GATCGCTCGTTCGTTGTTC-3’	124
	Reverse	5’-TCGCCTGAGGTTATCGTTT-3’	
waal	Forward	5'-GGATAGTTAGTGGCGTTGC-3'	92
	Reverse	5'-GGAGTAGGGTTGCTCTGGT-3'	

Supplementary Tab. 9 Primers for *waal* PCR in different clinical isolated strains

Strain	Forward/ Reverse	Primers (5' to 3')	bp
E. coli	*waal*-F	GCTTATTGTCACTAATTTTAC	21
	waal-R	CAGACCTTTCAGTGACCCTG	20
K. pneumonia	*waal*-F	GTCACTTAAACCATAGATAA	20
	waal-R	ACTCATAGCGAATATTGGTC	20
P. aeruginosa	*waal*-F	ACACTCGAGCAGCTCCTCAA	20
	waal-R	CGGCTGTTTTTCTCCTGATC	20

Supplementary Discussion

Notably, other metabolites like alanine, fructose, glucose, and glutamine promote the membrane potential, but they fail to reverse serum resistance. This discrepancy is attributed to the dual roles of glycine that not only promotes membrane potential but also up-regulates the expression of complement-binding outer membrane proteins. The proposed mechanism can be applicable in Gram-negative bacteria with outer membrane proteins in addition to *E. coli,* but why this effect is also observed in Gram-positive bacteria requires further investigation as they do not possess the outer membrane proteins for MAC insertion. The increased deposition of complement components on MRSA surface suggests other mechanisms may exist. Nevertheless, that the triple mutations of *htrE, nfrA* and *yhcD* did not completely abrogate glycine-mediated serum killing and loss of *waal* affected the killing potential in several mutants indicates that serum resistance is a multi-factorial event that may include more than membrane potential and outer membrane proteins.

Because complement-dependent killing represents a crucial innate immune response with a rapid but persistent mechanism after bacterial infection[1-3], glycine potentiation of complement/serum-dependent cell death has a potential clinical use. Most significantly, glycine could be used to potentiate the innate complement response in human patients who lack a strong acquired immune response, such as AIDS patients, or to protect immune-compromised individuals suffering from cystic fibrosis, healthcare-associated pneumonia, and ventilator-associated pneumonia. The mortality

rate from community-acquired bacterial bloodstream infections is as high as 46% in AIDS patients[4,5] and *P. aeruginosa* cause severe respiratory tract and systemic infections[6] and are a major cause of mortality and morbidity in immune-compromised individuals in hospital intensive care units[7]. Equally, the approach is also suitable in animal breeding and aquaculture since the glycine-enabled killing of bacterial pathogens by serum is detected in non-mammalian species, and glycine is used as nutrition for these animals. Finally, exogenous glycine potentiates ampicillin to kill serum-susceptible or serum-resistant and multidrug-resistant pathogens, which may be related to that exogenous glycine fuels the TCA cycle since the inhibited TCA cycle has been determined as a characteristic feature of antibiotic-resistant bacteria[8, 9, 10-17]. However, further mechanisms wait investigation.

Previous studies showed the role of glycine on cell wall composition and the growth of Gram-positive bacteria[18]. To exclude the possibility that glycine might alter the integrity of the cell wall of Gram-negative bacteria and thereby made bacteria sensitive to serum, antibiotic attack assay was performed. As shown in (**Supplementary Fig. 5c)**, metabolites or antibiotics alone did not kill the bacteria. More importantly, glycine did not increase the efficacy of antibiotics to kill the bacteria in the same buffer as glycine potentiated serum, indicating the unaffected integrity of the cell wall. The synergistic effect of glucose and antibiotics in killing bacteria was served as a positive control, which was shown previously[8, 19]. Instead, the present study indicates that glycine promotes the expression of

complement-binding proteins and membrane potential, which enabled the bacteria-killing by serum. In addition, since serum-sensitive bacteria are not necessarily killed by the MAC but by other non-complement heat-sensitive factors[20], C3-depleted serum and EDTA/EGTA-treated human serum were used to confirm that serum complement system contributes the glycine-enabled killing.

It is clear that multidrug-resistant and serum-resistant pathogens are a serious threat to human health and agriculture[21, 22]. The US Centers for Disease Control and Prevention has recently warned that drug-resistant bacteria kill at least 23,000 people annually in America and cost the US health-care system 20 billion dollars annually[23]. Therefore, it is encouraging that elevation of glycine, serine and threonine catabolic pathway to the TCA cycle potentiates serum-dependent death of multiple strains of Gram-negative and Gram-positive bacteria, via a mechanism that appears to be conserved in humans and other species. We are hopeful that the findings presented in this study will reduce the threat of infection with multidrug-resistant pathogens worldwide and will have broad application in medicine, agriculture and animal husbandry.

To exclude the possibility that downregulation of outer membrane proteins, including those which are known to interact with complement regulators, e.g. OmpA and OmpC, by exogenous glycine would also increase serum susceptibility, we detected percent survival of *ompA*- and *ompC*-deleted mutants, and found that loss of *ompA* and *ompC* led to lower percent survival in the presence of serum but not in the presence of serum

and glycine

In addition, it should be noted that mutants from the KEIO collection may cause phenotypes that are off-targets. And the knockouts may cause a metabolic shift, which is not necessarily related to the observed phenotype, through targeting specific metabolic genes. However, the present study utilizes a large set of mutants as well as other coincided experiments, and thereby ensures the reliability of these findings.

In summary, the present study discloses the regulation of glycine, serine and threonine catabolism to bacterial serum resistance and develops a metabolome-reprogramming strategy to revert serum-resistant metabolome to serum-sensitive metabolome, leading to the elimination of serum resistance. Out of the reprogramming metabolites, glycine and serine are screened. Interestingly, the mechanisms of the regulation not only attribute to metabolic flux, but also depend on substrate activation to enzymes, which lead to disclose of two unknown findings: the GlyA activation caused by high dose of glycine, serine and threonine surpasses over the negative regulation of PurR to *glyA* and *kbl* promoters, and a new metabolic regulation pathway from glycine to ATP synthase. At last, our developed approach highlights the way to control pathogens through reprogramming microbes' sensitivity to host immune defense.

Supplementary References

1. Kolev, M., Kemper, C. Keeping it all going-complement meets metabolism. *Front Immunol.* **8**, 1 (2017).

2. Maruvada, R., Blom, A.M., and Prasadarao, N.V. Effects of complement regulators bound to *Escherichia coli* K1 and Group B Streptococcus on the interaction with host cells. *Immunology* **124**, 265-276 (2008).

3. Wang, C.Y., Wang, S.W., Huang, W.C., Kim, K.S., Chang, N.S., Wang, Y.H., Wu, M.H., & Teng, C.H. Prc contributes to *Escherichia coli* evasion of classical complement-mediated serum killing. *Infect. Immun.* **80**, 3399-3409 (2012).

4. Raffatellu, M., Santos, R.L., Verhoeven, D.E., George, M.D., Wilson, R.P., Winter, S.E., Godinez, I., Sankaran, S., Paixao, T.A., and Gordon, M.A. Simian immunodeficiency virus-induced mucosal interleukin-17 deficiency promotes *Salmonella* dissemination from the gut. *Nat. Med.* **14**, 421-428 (2008).

5. Huson, M.A., Stolp, S.M., van der Poll, T., and Grobusch, M.P. Community-acquired bacterial bloodstream infections in HIV-infected patients: a systematic review. *Clin. Infect. Dis*. *5***8**, 79-92(2014).

6. Zhang, Y., Li, X., Carpinteiro, A., Goettel, J.A., Soddemann, M., and Gulbins, E. Kinase suppressor of Ras-1 protects against pulmonary *Pseudomonas aeruginosa* infections. *Nat. Med.* **17**, 341-346(2011).

7. Dettman, J.R., Rodrigue, N., Aaron, S.D., and Kassen, R. Evolutionary genomics of epidemic and nonepidemic strains of *Pseudomonas aeruginosa*. *Proc. Natl. Acad. Sci.USA* **110**, 21065 -21070(2013).

8. Peng, B., et al., Exogenous alanine or/and glucose plus kanamycin kills antibiotic-resistant bacteria. *Cell Metab* **21**, 249–261 (2015).

9. Su, Y.B., Peng, B., Han, Y., Li, H., & Peng, X.X. Fructose restores susceptibility of multidrug-resistant *Edwardsiella tarda* to kanamycin. *J Proteome Res* **14**, 1612-1620 (2015).

10. Kohanski, M.A., Dwyer, D.J., Hayete, B., Lawrence, C.A,, and Collins, J.J. A common mechanism of cellular death induced by bactericidal antibiotics. *Cell* **130**, 797-810(2007).

11. Kohanski, M.A., Dwyer. D.J., Wierzbowski, J., Cottarel, G., and Collins, J.J. Mistranslation of membrane proteins and two-component system activation trigger antibiotic-mediated cell death. *Cell* **135**, 679-690 (2008).

12. Kohanski, M.A., Dwyer, D.J., and Collins, J.J. How antibiotics kill bacteria: from targets to networks. *Nat. Rev. Microbiol*. **8**, 423-435 (2010).

13. Lin, X.M., Kang, L.Q., Li, H., and Peng, X.X. Fluctuation of multiple metabolic pathways is required for *Escherichia coli* in response to chlortetracycline stress. *Mol. Biosyst*. **10**, 901-908 (2014).

14. Purohit, H.J., Cheem, S., Lal, S., Raut, C.P., Kalia, V.C. In search of drug targets for *Mycobacterium tuberculosis*. *Infect Disord Drug Targets. 7,* 245-250 (2007).

15. Belenky, P., Ye, J.D., Porter, C.B., Cohen, N.R., Lobritz, M.A., Ferrante, T., Jain, S., Korry, B.J., Schwarz, E.G., Walker, G.C., Collins, J.J. Bactericidal antibiotics induce toxic metabolic perturbations that lead to cellular damage. *Cell Rep*. **13**,

968-80 (2015).

16. Liu, S.R., Peng, X.X., Li, H. Metabolic mechanism of ceftazidime resistance in *Vibrio alginolyticus*. *Infect Drug Resist.* **12**, 417-429 (2019).
17. Cheng, Z.X., Yang, M.J., Peng, B., Peng, X.X., Lin. X.M., Li, H. The depressing central carbon and energy metabolisms mediate levofloxacin resistance in *Vibrio alginolyticus*. *J Proteomics* **181**, 83-91 (2018).
18. Hammes, W., Schleifer, K.H., & Kandler, O. Mode of action of glycine on the biosynthesis of peptidoglycan. *J Bacteriol.* **116**, 1029-1053(1973).
19. Allison, K. R., Brynildsen, M. P. & Collins, J. J. Metabolite-enabled eradication of bacterial persisters by aminoglycosides. *Nature* **473**, 216-220 (2011).
20. Berends, E.T., Mohan, S., Miellet, W.R., Ruyken, M., Rooijakkers, S.H. Contribution of the complement membrane attack complex to the bactericidal activity of human serum. *Mol Immunol*. **65**, 328-335 (2015).
21. Zowawi, H.M., et al. The emerging threat of multidrug-resistant Gram-negative bacteria in urology. *Nat Rev Urol*. **12**, 570-584 (2015).
22. Tadesse, D.A., Zhao, S., Tong, E., Ayers, S., Singh, A., Bartholomew, M.J., McDermott, P.F. Antimicrobial drug resistance in *Escherichia coli* from humans and food animals, United States, 1950-2002. *Emerg. Infect. Dis.* **18**, 741-749 (2012).
23. McKenna, M. Antibiotic resistance: the last resort. *Nature* **499**, 394-396 (2013).
