# Supplementary material for: Glycine, serine and threonine metabolism confounds efficacy of complement-mediated killing
Source: Nat Commun. 2019 Jul 25;10:3325. doi: 10.1038/s41467-019-11129-5 (PMC6658569; doi:10.1038/s41467-019-11129-5)
Supplement: Supplementary file 3 — Reporting Summary [file 41467_2019_11129_MOESM3_ESM.pdf]

nature research

Corresponding author(s): Hui Li

Last updated by author(s): Jun 16, 2019

Reporting Summary

Nature Research wishes to improve the reproducibility of the work that we publish. This form provides structure for consistency and transparency in reporting. For further information on Nature Research policies, see Authors & Referees and the Editorial Policy Checklist.

Statistics

For all statistical analyses, confirm that the following items are present in the figure legend, table legend, main text, or Methods section.

n/a	Confirmed	
☐	☒	The exact sample size (*n*) for each experimental group/condition, given as a discrete number and unit of measurement
☐	☒	A statement on whether measurements were taken from distinct samples or whether the same sample was measured repeatedly
☐	☒	The statistical test(s) used AND whether they are one- or two-sided<br>*Only common tests should be described solely by name; describe more complex techniques in the Methods section.*
☐	☒	A description of all covariates tested
☐	☒	A description of any assumptions or corrections, such as tests of normality and adjustment for multiple comparisons
☐	☒	A full description of the statistical parameters including central tendency (e.g. means) or other basic estimates (e.g. regression coefficient) AND variation (e.g. standard deviation) or associated estimates of uncertainty (e.g. confidence intervals)
☐	☒	For null hypothesis testing, the test statistic (e.g. *F*, *t*, *r*) with confidence intervals, effect sizes, degrees of freedom and *P* value noted<br>*Give P values as exact values whenever suitable.*
☒	☐	For Bayesian analysis, information on the choice of priors and Markov chain Monte Carlo settings
☐	☒	For hierarchical and complex designs, identification of the appropriate level for tests and full reporting of outcomes
☐	☒	Estimates of effect sizes (e.g. Cohen's *d*, Pearson's *r*), indicating how they were calculated

Our web collection on statistics for biologists contains articles on many of the points above.

Software and code

Policy information about availability of computer code

Data collection	Peaks of total ion chromatograms (TIC) were identified by searching in MSD ChemStation (version E.02.02.1431, Agilent Technologies Inc., USA), and NIST MS Search 2.0 with NIST 2011 library (version 2.0, National Institute of Standards and Technology, Gaithersburg, MD).
Data analysis	Statistical analysis was performed in the SPSS 23.0. The difference among metabolites was conducted with ManneWhitney test (Wilcoxon rank sum test) and KruskaleWallis (KW) test. A 2-sided P value <0.05 was considered statistically significant. Both tests were non-parametric test which assumed the data was not normal distribution. Manne-Whitney U test judged the significance by the way of comparing the two samples' rank. False discovery rates (FDR) which indicated the proportion of true null hypotheses in the research were determined from q-value. The quantity π was another form of FDR and could be implemented in the R package "q-value". KW test was extension of Manne-Whitney U test. When significant result appeared in KW test, it showed at least one sample was different to others. Multiple testing didn't estimate the FDR. Z-score was used to analyze normalized area of differential metabolites. Z-score analysis scaled each metabolite according to a reference distribution. Samples from control were designated as the reference distribution. The mean and standard deviation of each metabolite were determined in these samples. Then, samples from in-infection were centered by the pre-infection mean and scaled by the pre-infection standard deviation. Orthogonal partial least square discriminant analysis (OPLS-DA) with software SIMCA 12.0 (Umetrics, Umeå, Sweden) was used to identify patterns associated with infection and minimize influence of the interindividual variation. Individuals with different phenotypes in the same group were termed interindividual variation. Data was log transformed prior to data import. Center (Ctr) scaling was selected before fitting. All variables were mean centered and scaled to standard deviation of each variable. Metabolic pathways are enriched by MetaboAnalyst 4.0 (

http://www.metaboanalyst.ca/)
Student's Test was used of SPSS statistics 13.0 software.

For manuscripts utilizing custom algorithms or software that are central to the research but not yet described in published literature, software must be made available to editors/reviewers. We strongly encourage code deposition in a community repository (e.g. GitHub). See the Nature Research guidelines for submitting code & software for further information.

Data

Policy information about availability of data

All manuscripts must include a data availability statement. This statement should provide the following information, where applicable:

- Accession codes, unique identifiers, or web links for publicly available datasets
- A list of figures that have associated raw data
- A description of any restrictions on data availability

Provide your data availability statement here.

Field-specific reporting

Please select the one below that is the best fit for your research. If you are not sure, read the appropriate sections before making your selection.

☒ Life sciences ☐ Behavioural & social sciences ☐ Ecological, evolutionary & environmental sciences

For a reference copy of the document with all sections, see nature.com/documents/nr-reporting-summary-flat.pdf

Life sciences study design

All studies must disclose on these points even when the disclosure is negative.

Sample size	For metabolomics analysis, six biologically equivalent samples and two technical replicas were prepared. Since three biological samples and two technical replicas are generally adopted in GC-MS based metabolomics, the current study increased the samples by twice. For the other experiments, data are representative of three independent biological replicates, which are widely accepted
Data exclusions	No data were excluded.
Replication	All attempts at replication were successful.
Randomization	Samples for biological repeats were randomly selected and grouped. Mice were randomly grouped.
Blinding	GC-MS based metabolomics and all in vivo studies was blind for data acquisition and analysis.

Reporting for specific materials, systems and methods

We require information from authors about some types of materials, experimental systems and methods used in many studies. Here, indicate whether each material, system or method listed is relevant to your study. If you are not sure if a list item applies to your research, read the appropriate section before selecting a response.

Materials & experimental systems

n/a	Involved in the study	
☐	☒	Antibodies
☐	☒	Eukaryotic cell lines
☒	☐	Palaeontology
☒	☐	Animals and other organisms
☐	☒	Human research participants
☐	☒	Clinical data

Methods

n/a	Involved in the study	
☐	☒	ChIP-seq
☒	☐	Flow cytometry
☐	☒	MRI-based neuroimaging

Antibodies

Antibodies used	C9 neoantigen (Catalog number: HM2167F (Human, mAB, aE11)) and C3b/iC3b antibody (Catalog number: HM22286 (Human, mAB, 3E7)) from Hycult Biotech Inc., Nederland. GlyA, Kbl, PurD, Crp, HtrE, YhcD, NfrA rabbit polyclonal antibody from our own preparation, which were validated by mutants. HRP, goat anti-rabbit IgG (Catalog number : 07062301) from C9 neoantigen (Catalog number: HM2167F (Human, mAB, aE11)) and C3b/iC3b antibody (Catalog number: HM22286 (Human, mAB, 3E7)) from Hycult Biotech Inc., Nederland. GlyA,

	Kbl, PurD, Crp, HtrE, YhcD, NfrA rabbit polyclonal antibody from our own preparation, which were validated by mutants. HRP, goat anti-rabbit IgG (Catalog number : 07062301) from C9 neoantigen (Catalog number: HM2167F (Human, mAB, aE11)) and C3b/iC3b antibody (Catalog number: HM22286 (Human, mAB, 3E7)) from Hycult Biotech Inc., Nederland. GlyA, Kbl, PurD, Crp, HtrE, YhcD, NfrA rabbit polyclonal antibody from our own preparation, which were validated by mutants. HRP, goat anti-rabbit IgG (Catalog number : 07062301) from Xiamen Bosheng Biotech Inc., Xiamen. Diagnostic sera for Entero-toxigenic Escherichia coli (Cat. TR301, TR302 & TR303) were commercially obtained from Ningbo Tianrun Bio-pharmaceutical Co. LTD, Ningbo, China. Anti-C3 Rabbit pAb（Cat. 381678), anti-C5 (N-term) Rabbit pAb (Cat. 616722), and Fluorescein (FITC) affiniPure goat anti-rabbit IgG (Cat.511201) were from Zenbio, China.
Validation	Nederland. GlyA, Kbl, PurD, Crp, HtrE, YhcD, NfrA rabbit polyclonal antibody from our own preparation, which were validated by mutants of these protein genes.

Eukaryotic cell lines

Policy information about cell lines

Cell line source(s)	*State the source of each cell line used.*
Authentication	*Describe the authentication procedures for each cell line used OR declare that none of the cell lines used were authenticated.*
Mycoplasma contamination	*Confirm that all cell lines tested negative for mycoplasma contamination OR describe the results of the testing for mycoplasma contamination OR declare that the cell lines were not tested for mycoplasma contamination.*
Commonly misidentified lines (See ICLAC register)	*Name any commonly misidentified cell lines used in the study and provide a rationale for their use.*

Human research participants

Policy information about studies involving human research participants

Population characteristics	The human serum was prepared from normal human adult during annual health examination.
Recruitment	Each individual signed the consent form that informs the use of the serum for research purpose.
Ethics oversight	The project is approved by the Institutional Animal Care and Use Committee of Sun Yat-sen University.

Note that full information on the approval of the study protocol must also be provided in the manuscript.

Clinical data

Policy information about clinical studies

All manuscripts should comply with the ICMJE guidelines for publication of clinical research and a completed CONSORT checklist must be included with all submissions.

Clinical trial registration	*Provide the trial registration number from ClinicalTrials.gov or an equivalent agency.*
Study protocol	*Note where the full trial protocol can be accessed OR if not available, explain why.*
Data collection	*Describe the settings and locales of data collection, noting the time periods of recruitment and data collection.*
Outcomes	*Describe how you pre-defined primary and secondary outcome measures and how you assessed these measures.*

ChIP-seq

Data deposition

- [ ] Confirm that both raw and final processed data have been deposited in a public database such as GEO.
- [ ] Confirm that you have deposited or provided access to graph files (e.g. BED files) for the called peaks.

Data access links *May remain private before publication.*	*For "Initial submission" or "Revised version" documents, provide reviewer access links. For your "Final submission" document, provide a link to the deposited data.*
Files in database submission	*Provide a list of all files available in the database submission.*

Genome browser session
(e.g. UCSC)

Provide a link to an anonymized genome browser session for "Initial submission" and "Revised version" documents only, to enable peer review. Write "no longer applicable" for "Final submission" documents.

Methodology

Replicates

Describe the experimental replicates, specifying number, type and replicate agreement.

Sequencing depth

Describe the sequencing depth for each experiment, providing the total number of reads, uniquely mapped reads, length of reads and whether they were paired- or single-end.

Antibodies

Describe the antibodies used for the ChIP-seq experiments; as applicable, provide supplier name, catalog number, clone name, and lot number.

Peak calling parameters

Specify the command line program and parameters used for read mapping and peak calling, including the ChIP, control and index files used.

Data quality

Describe the methods used to ensure data quality in full detail, including how many peaks are at FDR 5% and above 5-fold enrichment.

Software

Describe the software used to collect and analyze the ChIP-seq data. For custom code that has been deposited into a community repository, provide accession details.

Magnetic resonance imaging

Experimental design

Design type

Indicate task or resting state; event-related or block design.

Design specifications

Specify the number of blocks, trials or experimental units per session and/or subject, and specify the length of each trial or block (if trials are blocked) and interval between trials.

Behavioral performance measures

State number and/or type of variables recorded (e.g. correct button press, response time) and what statistics were used to establish that the subjects were performing the task as expected (e.g. mean, range, and/or standard deviation across subjects).

Acquisition

Imaging type(s)

Specify: functional, structural, diffusion, perfusion.

Field strength

Specify in Tesla

Sequence & imaging parameters

Specify the pulse sequence type (gradient echo, spin echo, etc.), imaging type (EPI, spiral, etc.), field of view, matrix size, slice thickness, orientation and TE/TR/flip angle.

Area of acquisition

State whether a whole brain scan was used OR define the area of acquisition, describing how the region was determined.

Diffusion MRI ☐ Used ☐ Not used

Preprocessing

Preprocessing software

Provide detail on software version and revision number and on specific parameters (model/functions, brain extraction, segmentation, smoothing kernel size, etc.).

Normalization

If data were normalized/standardized, describe the approach(es): specify linear or non-linear and define image types used for transformation OR indicate that data were not normalized and explain rationale for lack of normalization.

Normalization template

Describe the template used for normalization/transformation, specifying subject space or group standardized space (e.g. original Talairach, MNI305, ICBM152) OR indicate that the data were not normalized.

Noise and artifact removal

Describe your procedure(s) for artifact and structured noise removal, specifying motion parameters, tissue signals and physiological signals (heart rate, respiration).

Volume censoring

Define your software and/or method and criteria for volume censoring, and state the extent of such censoring.

Statistical modeling & inference

Model type and settings

Specify type (mass univariate, multivariate, RSA, predictive, etc.) and describe essential details of the model at the first and second levels (e.g. fixed, random or mixed effects; drift or auto-correlation).

Effect(s) tested

Define precise effect in terms of the task or stimulus conditions instead of psychological concepts and indicate whether ANOVA or factorial designs were used.

Specify type of analysis: ☐ Whole brain ☐ ROI-based ☐ Both

Statistic type for inference (See Eklund et al. 2016)	*Specify voxel-wise or cluster-wise and report all relevant parameters for cluster-wise methods.*
Correction	*Describe the type of correction and how it is obtained for multiple comparisons (e.g. FWE, FDR, permutation or Monte Carlo).*

Models & analysis

n/a	Involved in the study
☐	☐ Functional and/or effective connectivity
☐	☐ Graph analysis
☐	☐ Multivariate modeling or predictive analysis

Functional and/or effective connectivity	*Report the measures of dependence used and the model details (e.g. Pearson correlation, partial correlation, mutual information).*
Graph analysis	*Report the dependent variable and connectivity measure, specifying weighted graph or binarized graph, subject- or group-level, and the global and/or node summaries used (e.g. clustering coefficient, efficiency, etc.).*
Multivariate modeling and predictive analysis	*Specify independent variables, features extraction and dimension reduction, model, training and evaluation metrics.*
